# Evidence for Electrocardiographic Patterns Identifying Acute Coronary Occlusion in Non‐ST‐Elevation Acute Coronary Syndromes: A Scoping Review

**DOI:** 10.1111/acem.70389

**Published:** 2026-08-02

**Authors:** Andrew P. Grimes, Sonja J. Maria

**Affiliations:** ^1^ School of Nursing, Paramedicine and Healthcare Sciences Charles Sturt University Bathurst New South Wales Australia

**Keywords:** acute coronary syndrome, diagnostic accuracy, electrocardiography, occlusion myocardial infarction

## Abstract

**Background:**

Approximately one‐quarter to one‐third of patients with non‐ST‐elevation myocardial infarction have a completely occluded culprit coronary artery but are not routinely referred for emergent reperfusion. A growing literature describes electrocardiographic patterns purported to identify acute coronary occlusion (ACO) in non‐STEMI populations, but guideline adoption has been limited and inconsistent. We conducted a scoping review to map the extent, characteristics, and methodologies of primary studies examining non‐ST‐elevation ECG patterns against reference standards for ACO.

**Methods:**

Electronic databases were searched and supplemented by backward and forward citation tracking. Primary studies were included evaluating non‐ST‐elevation ECG patterns against an angiographic or composite reference standard for ACO. Studies using stenosis severity or anatomical disease burden as the reference standard were excluded.

**Results:**

Forty‐two studies were included, yielding 78 pattern‐level data sources spanning 20 ECG patterns. Only 23/78 data sources evaluated undifferentiated ACS patients. Measurable index test definitions were present in 33/78 data sources. Reference standards were highly heterogeneous. Although 70/78 data sources defined a reference standard for ACO, only 29/78 reported diagnostic test accuracy values, most of which were in the LBBB/VPR category.

**Conclusions:**

The literature is heterogeneous, methodologically uneven, and concentrated in a small number of pattern categories. Outside the LBBB/VPR criteria, diagnostic test accuracy data in NSTE‐ACS populations are sparse, and methodological features that critically affect their interpretation are inconsistently reported. These findings identify substantial gaps in the primary literature and inform the scope of future pattern‐specific diagnostic test accuracy systematic reviews.

## 1 Introduction

### 1.1 Rationale

The 12‐lead electrocardiogram (ECG) is the cornerstone of initial evaluation in suspected acute coronary syndrome (ACS). Guidelines recommend that patients presenting with symptoms suggestive of ACS should have a diagnostic 12‐lead ECG obtained and interpreted within 10 min of first medical contact, primarily assessing for electrocardiographic ST‐elevation myocardial infarction (STEMI) criteria [1, 2, 3]. Those meeting STEMI criteria should receive immediate reperfusion with fibrinolytic therapy and/or primary percutaneous intervention within 2 h, while those without sufficient ST‐elevation are categorized under non‐ST‐elevation acute coronary syndrome (NSTE‐ACS) [1, 2, 3].

This fundamentally dichotomous approach to ACS management is based on the prominent assertion that STEMI typically represents a completely occluded coronary artery producing ongoing transmural ischemia and infarction, while non‐STEMI (NSTEMI) represents a non‐occluded or partially occluded artery [1, 3]. However, the presence or absence of ST‐elevation is an inadequate proxy for epicardial coronary artery patency, and the weak evidence basis for this has been acknowledged for decades [4, 5].

In terms of diagnostic test accuracy, STEMI criteria are a poor rule‐out test for acute coronary occlusion (ACO). STEMI criteria have a specificity of 63%–97% but a sensitivity of only 44%–72%, and approximately one quarter to one third of NSTEMI patients have completely occluded culprit arteries [6, 7, 8, 9]. Although STEMI criteria are not used to rule out acute myocardial infarction, NSTE‐ACS patients are treated as ruled out for immediate reperfusion with few exceptions. As a result, patients with false‐negative STEMI criteria—those with occluded culprit arteries who do not meet electrocardiographic ST‐elevation criteria—do not receive timely reperfusion therapy [10].

Within 1–4 h of coronary artery occlusion, a wavefront of irreversible cardiomyocyte necrosis progresses through increasingly severe stages of tissue injury; with each stage, prognosis steeply worsens [11]. Reperfusion within these first hours can halt this progression, drastically reducing morbidity and mortality. Although guidelines recommend that NSTE‐ACS patients with refractory angina or hemodynamic or electrical instability should be referred for immediate reperfusion regardless of ECG findings, this recommendation is rarely followed [12, 13]. In practice, the mean time to angiography for NSTEMI patients with totally occluded culprit arteries is more than 30 h and is associated with significant rates of reinfarction, cardiogenic shock, acute left ventricular systolic dysfunction, and early all‐cause mortality [8, 9].

A growing, evidence‐informed understanding of false‐negative STEMI criteria and its impact on patient outcomes has driven calls to abandon or de‐emphasize the STEMI/NSTEMI paradigm in favor of ACS management based on underlying pathology and an expanded diagnostic approach [4, 11]. The preferred term, “occlusion myocardial infarction” (OMI), has been variably defined as “acute coronary occlusion or near occlusion with insufficient collateral circulation, such that downstream myocardium will undergo imminent necrosis without reperfusion” [14].

The proposition that ECG identification of ACO can improve meaningfully when expanded beyond STEMI criteria is no longer hypothetical; substantial research has demonstrated that human ECG experts and machine learning models can reliably outperform STEMI criteria and usual care [14, 15, 16]. However, translation of this research has been limited; specific “STEMI‐equivalent” ECG patterns have been adopted with significant variability in guidelines. This highlights a research gap, as studies informing the diagnostic accuracy of specific ECG patterns are fragmented and rarely synthesized.

We therefore conducted a review to map the extent, characteristics, and methodologies of existing primary studies examining non‐ST‐elevation ECG patterns associated with ACO. Scoping review methodology was determined to be most appropriate to characterize the breadth and methodological features of a heterogeneous literature [17]. We conducted a preliminary search of MEDLINE, the Cochrane Database of Systematic Reviews, JBI Evidence Synthesis, Open Science Framework, PROSPERO, and Epistemonikos in February 2026 to identify current or underway systematic reviews or scoping reviews matching our objectives. None were identified.

### 1.2 Objectives

#### 1.2.1 Review Question

What primary studies have examined the association between specific non‐ST‐elevation ECG patterns and acute coronary occlusion in patients with suspected or confirmed NSTE‐ACS?

#### 1.2.2 Population

Patients with suspected or confirmed NSTE‐ACS.

#### 1.2.3 Concept

Associations between ECG patterns, other than STEMI criteria, and acute coronary occlusion.

#### 1.2.4 Context

Settings where ECG interpretation guides reperfusion decisions.

#### 1.2.5 Types of Sources

Primary empirical studies. Case reports and case series were excluded.

## 2 Methods

This scoping review was conducted in accordance with the Joanna Briggs Institute (JBI) methodology for scoping reviews and is reported according to the Preferred Reporting Items for Systematic Reviews and Meta‐Analyses (PRISMA) extension for scoping reviews [18, 19]. The protocol is registered with Open Science Framework (https://osf.io/48wrh/).

### 2.1 Eligibility Criteria

ECG patterns that are purported to represent potential ACO were identified during the preliminary literature review, and the list refined iteratively during screening, per JBI guidance. Specific patterns and their varying operational definitions were placed in the following final categories: left bundle branch block (LBBB) and ventricular paced rhythm (VPR) criteria, posterior OMI, hyperacute T waves, de Winter T waves, Wellens syndrome, ST‐elevation in aVR patterns, subtle ST changes, and other (South African Flag, Aslanger pattern, precordial swirl).

Primary empirical studies were included if they reported on the specified ECG patterns in patients with suspected or confirmed ACS, against an angiographic or composite reference standard. Reference standard heterogeneity was anticipated during the preliminary literature review, as there is poor correlation between the severity of occlusion at the time of ECG and angiographic findings, and a “gold standard” reference test for ACO is not yet established [20, 21]. Accordingly, modern studies increasingly employ a composite reference standard that includes a combination of angiographic findings, highly elevated biomarkers, and echocardiographic regional wall motion abnormalities. As our purpose was to map these differences rather than adjudicate them, we included studies where the reference standard confirmed the presence of ACO, whether reported as angiographic TIMI flow, total/complete occlusion, or a composite standard incorporating angiographic data with biomarker or echocardiographic confirmation.

Studies were excluded where the reference standard is limited to clinical features, prognostic outcomes, or where angiographic reporting is limited to stenosis severity or disease burden without confirmation of occlusion status or TIMI flow. There is currently no guideline basis for emergent reperfusion triggered by stenosis severity or left main/triple vessel disease (LM/3VD) anatomy. Finally, data derived from mixed STEMI/NSTEMI populations were not charted, consistent with the review's concept.

### 2.2 Search

A three‐step search strategy following JBI guidance [22] was developed by the primary author with assistance from two librarians. The search strategy was adapted for MEDLINE, Embase, Scopus, Cochrane Library, ProQuest Dissertations and Theses Global, medRxiv, World Health Organization International Clinical Trials Registry Platform, and Open Science Framework (OSF). Google Scholar was searched in a logged‐out browser session to minimize result personalisation [23]. Screening of this source was limited to the first 200 results sorted by relevance, consistent with the approach reported in Cochrane Reviews [24]. We performed a supplemental search in Medline, Embase, and Scopus to identify interpreter studies with relevant pattern‐level data; this was not planned in the original protocol. The full record of search strategies is reported in Appendix S1. Finally, backward and forward citation searching was undertaken for included studies and relevant review and background papers. Although citation searching of case studies was planned in the original protocol, the number of case studies identified during screening was substantially greater than anticipated, rendering citation searching of case studies impractical.

### 2.3 Selection of Sources of Evidence

All search results were subject to the same screening process. Duplicates were removed in Covidence. Following a pilot test, titles and abstracts were screened independently by two reviewers. Potentially relevant sources were assessed in detail by two independent reviewers. Disagreements were resolved at each stage through discussion. Reasons for exclusion of full‐text sources are reported in Appendix S2.

Sources that reached full‐text screening but for which no full text could be located, including through direct contact with authors, were excluded with the reason recorded as “no full text available.” Where a potentially includable source was not available in English, the study was translated to English using a large language model (LLM), an accepted and increasingly common practice in scoping reviews [25]. Two large language models (Claude Opus 4.7 and Claude Sonnet 4.6; Anthropic) translated the non‐English papers independently and the translations were compared, finding no differences that would impact extraction. Five studies were translated this way with three included in the final results; these are reported in the Charting Dataset (see Data Availability Statement section).

### 2.4 Data Charting Process

Data from included studies were charted using a custom charting tool developed for this review. Charting was performed by the primary reviewer, with initial and regular auditing by a second reviewer. Discrepancies were resolved through discussion.

We charted data related to study design, index test definition, ECG interpretation, reference standards, timing, and results. Bibliographic and study‐level data were charted once per study. All subsequent data were charted at the level of the individual ECG pattern: the data source, defined as one study's data for a single pattern or variation, was the unit of extraction. A study reporting on a single pattern therefore yielded one data source, while a study reporting on multiple patterns or variations yielded several, each charted independently. Formal appraisal was not conducted. We charted study results for the purpose of mapping differences according to methodological features, but results were not pooled, synthesized, or used to draw conclusions about diagnostic test accuracy due to the scoping nature of this review [26].

## 3 Results

### 3.1 Selection of Sources of Evidence

**FIGURE 1:**
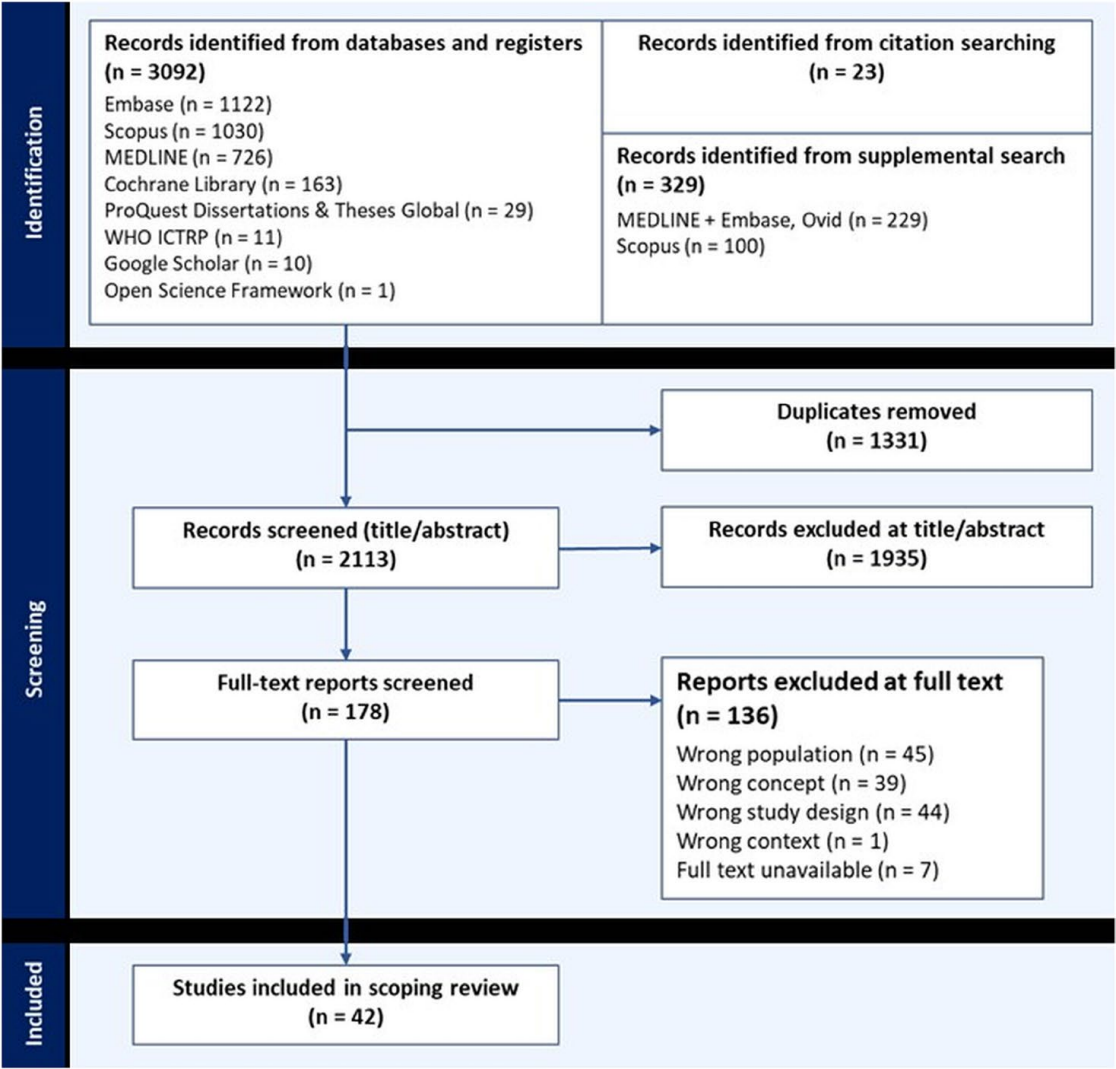
PRISMA flow diagram. ECG, electrocardiogram; PRISMA, Preferred Reporting Items for Systematic Reviews and Meta‐Analyses.

3444 records were identified through database searching and citation tracking (Figure 1). After removing duplicates, 1935 studies were excluded through title and abstract screening. Full text of 178 studies was screened, and 136 were excluded. 42 studies were included for charting, yielding 78 independent data sources.

### 3.2 Characteristics of Included Studies

Foundational characteristics of each included study are provided in Appendix S3. The complete set of data items charted from each study is provided in the Charting Dataset, available on the Open Science Framework (https://osf.io/w8347).

### 3.3 Study Design and Population Selection

46% of data sources examine confirmed NSTE‐ACS or high‐risk cohorts (e.g., STEMI activation networks, referred for angiography due to clinical features), and 24% include cohorts with angiographically confirmed ACO (Table 1). Only 23/78 (29%) data sources evaluate undifferentiated patients with suspected ACS, and 16/23 of those are in the LBBB/VPR criteria category.

**TABLE 1:** Population selection.

Pattern category	Data sources (*n*)^a^	Population selection		
		Undifferentiated/suspected ACS	Confirmed NSTE‐ACS or high‐risk cohort	Confirmed ACO
LBBB/VPR criteria	20	16/20	4/20	0/20
Posterior OMI	13	0/13	7/13	6/13
Hyperacute T waves	5	3/5	1/5	1/5
de Winter	9	0/9	4/9	5/9
Wellens syndrome	7	0/7	6/7	1/7
STE‐aVR patterns	7	0/7	7/7	0/7
Subtle ST changes	12	3/12	4/12	5/12
Aslanger pattern	1	1/1	0/1	0/1
Terminal QRS distortion	1	0/1	0/1	1/1
Precordial swirl	3	0/3	3/3	0/3
Total	78	23/78 (29%)	36/78 (46%)	19/78 (24%)

Abbreviations: LBBB, left bundle branch block; VPR, ventricular paced rhythm.

^a^ Data sources reflect distinct pattern‐level extractions. Some studies contribute to multiple categories.

### 3.4 Index Test Reproducibility

Diagnostic test accuracy is sensitive to the effects of subjective interpretation and varying thresholds. Subjectivity increases the risk of multiple forms of bias, and threshold effects (variation between studies in what defines a positive result) can significantly alter the test's performance [27, 28]. We charted data for 20 specific ECG patterns (Table 2). Few patterns have consistent, established definitions. Many studies define the pattern by name only. In Table 2, an ECG pattern is considered measurable when each required component has a quantitative threshold (e.g., millimeters, ratios, scores), whereas non‐measurable definitions contain components described qualitatively (e.g., “tall”, “subtle”, “prominent”, “hyperacute”, “biphasic”) without a quantitative cutoff or threshold.

**TABLE 2:** Index test characteristics.

Pattern category	Specific pattern^a^	Data sources (*n*)	Sources with measurable index test definition
LBBB/VPR criteria	Smith‐Modified Sgarbossa	9	9/9
	Weighted Sgarbossa	4	4/4
	Unweighted Sgarbossa	4	4/4
	Selvester	2	2/2
	Barcelona	1	1/1
de Winter	de Winter pattern	9	0/9
Posterior OMI	Isolated anterior STD (V1–V3/4)	9	5/9
	Prominent R in V1	2	1/2
	Synthesized posterior STE	1	1/1
	STE in V7–V9	1	1/1
Wellens syndrome	Wellens pattern	7	0/7
Subtle ST changes	Subtle/sub‐threshold STE	8	0/8
	Isolated reciprocal STD	4	0/4
STE‐aVR patterns	STE‐aVR (±diffuse STD)	4	1/4
	Isolated STE‐aVR (post‐ROSC)	2	2/2
	STE‐aVR + STE in aVL	1	0/1
Hyperacute T waves	Hyperacute T waves	5	2/5
Other	Precordial swirl (variants 1–3)	3	0/3
	Terminal QRS distortion	1	0/1
	Aslanger pattern	1	0/1
Total		78	33/78 (42%)

Abbreviations: LBBB, left bundle branch block; OMI, occlusion myocardial infarction; ROSC, return of spontaneous circulation; STD, ST‐depression; STE, ST‐segment elevation; VPR, ventricular paced rhythm.

^a^ Complete per‐source operational definitions are presented in the Charting Dataset.

### 3.5 Reference Standards and Diagnostic Test Accuracy Values

Reference standards are grouped into three mutually exclusive categories: studies that define ACO using an angiographic or composite reference standard, and those that descriptively report TIMI flow grades without specifying which grades constitute ACO (Table 3). 70/78 (90%) data sources define a reference standard for ACO. However, only 29/78 (37%) report diagnostic test accuracy (DTA) values—sensitivity, specificity, predictive values, likelihood ratios, or odds ratios—and 20/29 (69%) of those that do are in the LBBB/VPR category. Only 9 sources, derived from 6 total studies, report DTA values for any other ECG pattern.

**TABLE 3:** Reference standard and DTA characteristics.

Category	Studies (*n*)	Data sources (*n*)*	Reference standard reporting			Sources reporting DTA values
			ACO/OMI reference standard		Descriptive TIMI flow grades only	
			Composite	Angiographic only		
LBBB/VPR criteria	9	20	15/20	5/20	0/20	20/20
Posterior OMI	10	13	2/13	11/13	0/13	4/13
Hyperacute T waves	4	5	3/5	2/5	0/5	2/5
de Winter	9	9	0/9	5/9	4/9	0/9
Wellens syndrome	7	7	0/7	4/7	3/7	0/7
STE‐aVR patterns	5	7	0/7	7/7	0/7	2/7
Subtle ST changes	8	12	4/12	7/12	1/12	1/12
Aslanger pattern	1	1	1/1	0/1	0/1	0/1
Terminal QRS distortion	1	1	0/1	1/1	0/1	0/1
Other/not classified	1	3	3/3	0/3	0/3	0/3
Total	42	78	28/78 (36%)	42/78 (54%)	8/78 (10%)	29/78 (37%)

* Data sources reflect distinct pattern‐level extractions. Some studies contribute to multiple categories. Abbreviations: ACO, acute coronary occlusion; DTA, diagnostic test accuracy; LBBB, left bundle branch block; OMI, occlusion myocardial infarction; STE, ST‐elevation; TIMI, thrombolysis in myocardial infarction; VPR, ventricular paced rhythm.

### 3.6 Pattern‐Level Findings

#### 3.6.1 Hyperacute T Waves

Abnormally large, symmetric T waves have been described as an early electrocardiographic sign of acute myocardial infarction for many decades [29, 30]. We identified only four studies includable for our review, with substantial author overlap. Three are interpreter studies with no operational pattern definition [15, 31, 32]. The fourth paper is a derivation and validation study reporting DTA values, including > 98% specificity of hyperacute T waves to identify ACO in a 1300‐patient validation cohort [33]. However, the index test requires proprietary software to measure.

#### 3.6.2 Subtle ST Changes

This category includes non‐consecutive or sub‐STEMI threshold ST‐elevation, reciprocal or isolated ST‐depression, and some combination patterns (e.g., inferior ST‐depression + ST‐elevation in aVL). This is the most heterogeneous category in our review; no two studies or data sources share both an index test definition and reference standard. No studies in this category include a measurable index test definition (primarily because they do not specify a minimum ST deviation), and only one study reports DTA values [34]. Our review identified no studies in this category that use an unconfirmed chest pain or NSTE‐ACS population with a reproducible or measurable pattern definition.

#### 3.6.3 de Winter Pattern

The peer‐reviewed literature on de Winter syndrome is dominated by case reports and case series, which were excluded from this review. A 2026 systematic scoping review of the pattern pooled 322 patients from 159 studies, of which 149 were case reports and 3 were case series [35]. Only nine primary studies met our inclusion criteria.

All definitions of de Winter pattern in our data include variably defined “upsloping” ST‐depression. No study defines the associated hyperacute T waves beyond “symmetric.” All sample sizes are small as there is a low prevalence of this pattern. This is despite prominent selection bias (all cohorts are confirmed NSTE‐ACS or angiographically confirmed occlusion), which tends to inflate prevalence. The study with the largest sample size in our data found 35 de Winter patients out of 1890 patients with confirmed anterior myocardial infarction, for 2% prevalence [36]. Across studies, the proportion of de Winter patients with TIMI 0–1 at angiography ranged from 50% to 100%, with no apparent grouping of results by study characteristics. We found no data on the prevalence or accuracy of the de Winter pattern in broader chest pain or NSTE‐ACS populations.

#### 3.6.4 Wellens Syndrome

By definition, Wellens syndrome presents after ischemic symptoms have resolved and is conventionally interpreted as evidence of reperfusion rather than active ACO [37]. However, the peer‐reviewed literature and guidelines are inconsistent regarding whether Wellens syndrome should be considered a STEMI‐equivalent, either due to ongoing occlusion or the imminent risk of re‐occlusion [1, 38, 39, 40]. We mapped studies meeting our inclusion criteria rather than adjudicate this controversy.

A significant number of search results were excluded as case studies, case series, or using stenosis‐only reference standards. Index test definitions for Wellens are consistent, with some disagreement about precordial lead distribution; however, none are fully measurable. All reference standards are purely angiographic.

Selection bias is prominent; all sources select populations with high‐risk features or confirmed coronary occlusions. One prospective study included a troponin‐negative population and performed angiography based on ischemic symptoms and the Wellens ECG pattern [41]. Despite the methodologically stronger design, their findings were consistent with the broader literature.

Across included studies, Wellens syndrome is described as representing a high degree of LAD stenosis; non‐LAD culprit arteries and/or occlusion (TIMI 0–1) at the time of angiography are consistently reported as rarities. The Wellens study with the largest sample size in our review reported a 96.5% specificity for identifying *non*‐occlusion at angiography [42]. The question of urgency in this population due to disease burden and the need for revascularization is outside the scope of this review.

#### 3.6.5 Posterior OMI

The standard 12‐lead ECG is poorly suited to detecting acute occlusion of arteries supplying the posterior (inferobasal) wall, which frequently produces transmural infarction without diagnostic ST‐elevation [8, 43].

ECG patterns are split into three categories: anterior ST‐depression (V1–V3/4), posterior ST‐elevation (V7–V9), and prominent R in V1. Most studies use a 0.5 mm cutoff for both anterior ST‐depression and posterior ST‐elevation, while some accept any magnitude of ST deviation. Only one study required a minimum 1 mm ST‐elevation in V7–V9 [44].

Only 4/13 data sources report any DTA values; 6/13 report only pattern prevalence in populations with confirmed occlusions. Two studies report sensitivity and specificity for ST‐depression in V1–V3/4 to identify TIMI 0–1 occlusion, with comparable findings showing sensitivity of 20%–27% and specificity of 92%–96% [42, 45].

Balloon occlusion studies, despite a methodologically superior reference standard, were excluded on population and context grounds. These studies consistently report ST‐elevation in V7–V9 during balloon occlusion of the left circumflex artery; anterior ST‐depression appears more variably and was absent in a substantial minority of cases demonstrating posterior ST‐elevation [46, 47, 48, 49]. A 2026 systematic review assessing the diagnostic accuracy of 12‐lead versus 18‐lead ECG similarly reported that approximately 20%–25% of posterior infarctions detected by ST‐elevation in V7–V9 did not demonstrate reciprocal anterior ST‐depression [50].

#### 3.6.6 STE‐aVR Patterns

ST‐elevation in lead aVR (STE‐aVR), typically with diffuse ST‐depression, commonly represents global subendocardial ischemia due to LM/3VD, but it is also produced by proximal LM or LAD obstruction, prompting debate about whether emergent reperfusion is indicated [51, 52, 53].

Survival bias is a concern in this literature, as proximal LM and LAD occlusions carry a high risk of death before angiography, and patients are not included in angiographic studies if they do not survive to reach the catheterization table [54, 55]. The prevalence of LM/LAD occlusion amongst patients with STE‐aVR in the prehospital and emergency department environments may therefore be systematically underestimated.

Our included studies describe generally poor correlation between STE‐aVR and ACO. One study stands out as an exception: Yamamoto et al. [56] evaluated cardiac arrest patients who had achieved return of spontaneous circulation (ROSC) and underwent coronary angiography due to a presumed cardiac cause of arrest, without diagnostic ST‐elevation on their initial post‐ROSC ECG. Of these, 27% had ACO, and 50% of those presented with persistent STE‐aVR on their follow‐up ECG (median 137 min after ROSC). For the follow‐up ECG, sensitivity was 50% and specificity 85% for ACO. Notably, the stronger STE‐aVR/ACO correlation reported in this cohort of resuscitated cardiac arrest patients, a population not well captured by in‐hospital cohorts, supports the survival bias concern raised above.

#### 3.6.7 Left Bundle Branch Block an d Ventricular Paced Rhythm Criteria

This category represents the largest and most methodically studied portion of our data. Eighty percent of the data sources use an undifferentiated cohort with suspected ACS and LBBB or VPR. All sources compare a fully measurable index test against an ACO definition and report DTA values. All studies report sensitivity and specificity, with most also reporting predictive values and likelihood ratios. Criteria are defined in their foundational studies and remain highly consistent; variation is limited to studies explicitly testing modifications.

Several criteria for identifying ACO in LBBB and/or VPR have been derived and tested (Table 4). These include the original weighted and unweighted Sgarbossa criteria, Smith‐Modified Sgarbossa criteria, Barcelona criteria, and Selvester criteria. Some guidelines consider the weighted Sgarbossa and Smith‐Modified Sgarbossa criteria to be STEMI‐equivalent, and a 2025 systematic review and meta‐analysis confirmed that the Smith‐Modified Sgarbossa criteria provide superior diagnostic accuracy in LBBB, substantially improving sensitivity over the original Sgarbossa criteria (84% vs. 40%) with minimal loss of specificity (93% vs. 97%) [38, 61].

**TABLE 4:** LBBB/VPR criteria.

Criteria	Definition
Unweighted Sgarbossa [57]	≥ 1 mm concordant STE in any lead, or ≥ 1 mm concordant STD in V1, V2 or V3, or Discordant STE > 5 mm
Weighted Sgarbossa [57]	STE ≥ 1 mm and concordant with QRS (5 pts), or STD ≥ 1 mm in V1, V2 or V3 (3 pts), or Discordant STE ≥ 5 mm (2 pts) Require ≥ 3 points to be positive
Smith‐Modified Sgarbossa [58, 59]	Concordant STE ≥ 1 mm, or Concordant STD ≥ 1 mm in V1–V3 (V1–V6 in VPR), or Discordant STE with ST/S ratio ≥ 25%
Barcelona [60]	Concordant STE ≥ 1 mm, or Concordant STD ≥ 1 mm (any lead), or Discordant ST deviation > 1 mm in any lead with dominant R or S < 6 mm
Selvester [60]	Concordant STE ≥ 1 mm, or STD ≥ 1 mm in V1–V3, or STE ≥ 2 mm + 10% of (S‐wave amplitude − R‐wave amplitude) in V2–V3, or STE ≥ 1 mm + 10% of (S‐wave amplitude − R‐wave amplitude) in remaining leads

Abbreviations: LBBB, left bundle branch block; QRS, QRS complex; STD, ST‐segment depression; STE, ST‐segment elevation; VPR, ventricular paced rhythm.

#### 3.6.8 Other Patterns

The terminal QRS distortion pattern was found in only one included data source, a study reporting that four of 20 patients with confirmed LAD occlusion not meeting STEMI criteria showed terminal QRS distortion [32].

For the Aslanger pattern, only the foundational study was included [62]. No foundational or primary studies were identified for South African Flag sign or Northern OMI.

One study evaluated the precordial swirl pattern [63]. Diagnostic accuracy figures are reported across shifting denominators, and the only findings reported in a STEMI‐negative group are sensitivity among those with LAD‐culprit ACO. The diagnostic performance of this pattern in NSTE‐ACS populations therefore remains uncharacterised.

## 4 Discussion

We charted author‐reported biases, and a summary of both reported and unreported biases in this literature is included in Appendix S4. Briefly, population selection and systematic features of STEMI pathways act as confounders across much of the literature, limiting internal validity.

### 4.1 Index Test and Reference Standard Conflation

Across the literature, the index test and reference standard are conflated in inconsistent and often contradictory ways. This was apparent from the preliminary literature review and persisted across many included studies.

- *Prospective diagnosis by ECG*: Patients receive an initial diagnosis of STEMI or NSTEMI according to their diagnostic ECG findings, and this is never changed regardless of angiographic or clinical outcomes. McLaren et al. [10] demonstrated this “no false negative paradox” directly, finding that 32% of patients with a discharge diagnosis of NSTEMI based on their diagnostic ECG met OMI criteria.
- *Retrospective diagnosis by angiography*: Conversely, we also observed that STEMI is often diagnosed retrospectively. Many of the studies we included or screened describe angiographically confirmed ACO populations as all‐STEMI cohorts, even when the population, or a subgroup within the population, did not have diagnostic ST‐elevation on their ECG.

This does not conflict with McLaren's findings but rather extends them. When the ECG or angiographic findings do not align with the STEMI/NSTEMI dichotomy, the diagnosis is either adjusted or preserved according to whichever fits the established paradigm.

### 4.2 Index Test Ambiguity

Many ECG patterns lack consistent definitions. Only 33/78 (42%) data sources in our review used measurable index test definitions, and only 13/58 (22%) were measurable outside of LBBB/VPR criteria. Fewer still report measurement methods such as whether the ST segment was measured at the J‐point or after, and whether it was compared with the T–P or P–Q segments.

Diagnostic accuracy can vary significantly with the test definition [64]. For example, the original weighted Sgarbossa criteria and the Smith‐Modified Sgarbossa criteria differ only in how the third criterion is applied: the original requires ≥ 5 mm of discordant ST‐elevation, while the modified version requires ST‐elevation ≥ 25% of the preceding S wave depth. This small change in the index test definition nearly doubles the sensitivity from 43% to 84% with minimal change in specificity [61]. This is a threshold effect; small differences in where the cutoff is set between positive and negative results can substantially shift sensitivity and specificity [28].

Undefined index tests require subjective interpretation. Although inter‐observer variability can be measured within studies, the diagnostic accuracy cannot be reliably translated to general practice without validation in those settings [64]. Most studies of ECG patterns for ACO identification have not defined the index test in a form that supports reliable translation to practice.

Mimics, conditions that produce ECG findings similar to the patterns under investigation, are not systematically characterized for most non‐STEMI patterns. STEMI has well‐recognized mimics including left ventricular hypertrophy, pericarditis, early repolarisation, hyperkalemia, and Brugada syndrome [65]. Awareness of mimics allows for clinical adjudication of false positives.

Future DTA research would benefit from establishing measurable ECG pattern definitions, along with systematic identification of mimics. This will reduce false positives, support evidence synthesis, and improve translation to clinical practice and guideline development.

### 4.3 Reference Standard Heterogeneity

The absence of a gold standard reference test for ACO is reflected in substantial heterogeneity across the literature. Reference standards differ across studies sharing common index test definitions, studies from overlapping research groups, and occasionally within individual studies. These differences complicate critical appraisal [66].

The interval between the index test and reference standard is also a major source of bias in DTA research, where longer delays introduce the effects of disease progression or treatment [66]. In ACS, those with STEMI receive immediate reperfusion, while in NSTEMI the time between the ECG and angiography can range from minutes to days. This effect is sometimes observable in the data; for example, Tang et al. [67] performed emergency angiography on de Winter patients as soon as they were identified and found 100% to have TIMI 0 flow, whereas in Wiśniewski et al. [34] only 60% of de Winter patients had TIMI 0 flow when angiography was performed within 24 h. While other factors likely contribute, this is consistent with disease progression or spontaneous reperfusion during the interval delay.

In our data, the index‐to‐reference interval was rarely reported (see Charting Dataset). Studies of populations referred for emergent reperfusion presumably aimed to perform angiography within 2 h per guidelines, but only one [68] reported the interval directly in any quantitative form.

### 4.4 Limitations

Three studies were translated by large language models (LLM). LLM translation fidelity for scoping reviews has not been established in the peer‐reviewed literature and is an active methodological question [25]. Although we independently translated each study using two different models, accuracy is not guaranteed. Proprietary LLMs are subject to frequent, undisclosed updates that can alter output behavior and limit the applicability of any validation of a specific model version [69].

Many studies did not specify how or whether acute occlusions were differentiated from chronic occlusions. We did not apply this differentiation as an inclusion criterion because doing so would have required a degree of adjudication inconsistent with the mapping aim of a scoping review, and because the information needed to make the distinction was not consistently reported. The extent to which chronic total occlusions may have been incorporated into the reference standards of individual studies is therefore unclear.

The inconsistency in terminology observed may have affected our screening process. Studies that retrospectively reclassified patients as STEMI based on angiographic findings, despite STEMI‐negative ECGs, may have been excluded as apparent all‐STEMI cohorts at title and abstract screening. The population inclusion criteria (suspected or confirmed NSTE‐ACS) were applied by two independent reviewers, but at the title and abstract stage this decision necessarily relied on how studies described their own populations.

## 5 Conclusions

The existing evidence on ECG patterns for identifying acute coronary occlusion in NSTE‐ACS patients is dominated by case studies, case series, and frequency or prevalence data amongst high‐risk populations or cohorts with confirmed occlusions. Only a small and largely recent subset of primary research adopts methodology consistent with diagnostic test accuracy studies, mostly limited to a few select ECG patterns.

Although it has been demonstrated that ECG experts and artificial intelligence can accurately identify ACO that is otherwise missed by STEMI criteria, the existing pattern‐level research is methodologically insufficient to support rigorous evidence synthesis and guideline adoption in most cases. For clinicians, this means that identification of ACO in the NSTE‐ACS population is conceptually supported, but there is fragmentary supporting evidence for how most individual ECG patterns are defined, distinguished from mimics, their diagnostic performance, or the appropriate degree of urgency.

This represents a substantial opportunity for future primary studies using methodology consistent with diagnostic test accuracy research to validate the individual ECG patterns through which expert interpreters identify ACO. Finally, a consensus reference standard for ACO is urgently needed.

## Author Contributions

A.P.G. contributed to study concept and design, acquisition of the data, analysis and interpretation of the data, drafting of the manuscript, and critical revision of the manuscript for important intellectual content. S.J.M. contributed to study concept and design, analysis and interpretation of the data, critical revision of the manuscript for important intellectual content, and provided study supervision. Both authors approved the final manuscript.

## Funding

The authors have nothing to report.

## Ethics Statement

The authors have nothing to report.

## Consent

The authors have nothing to report.

## Conflicts of Interest

The authors declare no conflicts of interest.

## Supporting information


**Appendix S1:** Search strategy.
**Appendix S2:** Sources excluded following full‐text review.
**Appendix S3:** Reference list.
**Appendix S4:** References.

## Data Availability

The Charting Dataset is openly available on OSF at https://osf.io/w8347.
