# Supplementary material for: Evidence for Electrocardiographic Patterns Identifying Acute Coronary Occlusion in Non‐ST‐Elevation Acute Coronary Syndromes: A Scoping Review
Source: Acad Emerg Med. 2026 Aug 2;33(8):e70389. doi: 10.1111/acem.70389 (PMC13429945; doi:10.1111/acem.70389)
Supplement: Supplementary file 1 — Appendix S1: Search strategy. Appendix S2: Sources excluded following full‐text review. Appendix S3: Reference list. Appendix S4: References. [file ACEM-33-0-s001.docx]

**Supplementary Methods and Data**

Supplemental Appendix S1. Search strategy

**Ovid Medline**March 17, 2026

| 1 | Myocardial Infarction/ or Anterior Wall Myocardial Infarction/ or Inferior Wall Myocardial Infarction/ or Non-ST Elevated Myocardial Infarction/ or ST Elevation Myocardial Infarction/ or coronary occlusion/ or coronary thrombosis/ | 205925 |
| --- | --- | --- |
| 2 | (myocardial infarction or STEMI or NSTEMI or non-stemi or elevation myocardial infarction or OMI or NOMI or occlusion myocardial infarction or occlusion MI or coronary occlusion or coronary artery occlusion).ti,ab. | 212514 |
| 3 | (hyperacute T wave* or symmetrical T wave* or prominent T wave* or posterior myocardial infarction or posterior MI or posterior STEMI or posterior wall myocardial infarction or posterior wall MI or posterior wall STEMI or posterior isch?emia or (ST adj2 depression adj5 (V1 or V2 or V3 or V4)) or (anterior ST adj2 depression) or Sgarbossa or (ST adj2 elevation adj4 aVR) or aVR sign or (diffuse ST adj2 depression) or (subtle ST adj2 elevation) or (minimal ST adj2 elevation) or (subthreshold ST adj2 elevation) or de Winter* or upsloping ST or up sloping ST or Wellen* or biphasic T wave* or deeply inverted T wave* or Aslanger* or South African flag or terminal QRS distortion or subtle anterior STEMI or (subtle anterior ST adj2 elevation) or STEMI equivalent).ti,ab. | 1636 |
| 4 | 1 or 2 | 283925 |
| 5 | 3 and 4 | 969 |
| 6 | 5 not (papillary muscle rupture or free wall rupture or ventricular septal rupture or cardiac rupture or mitral regurgitation or hernia or pancreatitis or mesenteric or dissection or ablation or covid or coronavirus or exercise or diabet* or stress test* or transplant or stroke or smoking or ticagrelor or clopidogrel or atorvastatin or sleep or pneumonia or sepsis).ti,ab. | 813 |
| 7 | 6 not “a case”.ti,ab. | 726 |

**Scopus**March 16, 2026

| # | Query | Results |
| --- | --- | --- |
| 1 | (TITLE-ABS-KEY("myocardial infarction" OR STEMI OR NSTEMI OR "non-stemi" OR "elevation myocardial infarction" OR OMI OR NOMI OR "occlusion myocardial infarction" OR "occlusion MI" OR "coronary occlusion" OR "coronary artery occlusion")) | 371695 |
| 2 | ⮑ AND (TITLE-ABS-KEY("hyperacute T wave*" OR "symmetrical T wave*" OR "prominent T wave*" OR "posterior myocardial infarction" OR "posterior MI" OR "posterior STEMI" OR "posterior wall myocardial infarction" OR "posterior wall MI" OR "posterior wall STEMI" OR "posterior ischemia" OR (ST PRE/2 depression PRE/5 (V1 OR V2 OR V3 OR V4)) OR (“anterior ST” PRE/2 depression) OR Sgarbossa OR (ST PRE/2 elevation PRE/4 aVR) OR "aVR sign" OR (diffuse ST PRE/2 depression) OR (subtle ST PRE/2 elevation) OR (“minimal ST” PRE/2 elevation) OR (“subthreshold ST” PRE/2 elevation) OR "de Winter*" OR "upsloping ST" OR "up sloping ST" OR Wellen* OR "biphasic T wave*" OR "deeply inverted T wave*" OR Aslanger* OR "South African flag" OR "terminal QRS distortion" OR "subtle anterior STEMI" OR ("subtle anterior ST" PRE/2 elevation) OR "STEMI equivalent*")) | 1759 |
| 3 | ⮑ AND NOT (TITLE-ABS-KEY("papillary muscle rupture" OR "free wall rupture" OR "ventricular septal rupture" OR "cardiac rupture" OR "mitral regurgitation" OR hernia OR pancreatitis OR mesenteric OR dissection OR ablation OR covid OR coronavirus OR exercise OR diabet* OR "stress test*" OR transplant OR atorvastatin OR "beta adrenergic receptor blocking agent" OR smoking)) | 1232 |
| 4 | ⮑ AND (LIMIT-TO(DOCTYPE,"ar") OR LIMIT-TO(DOCTYPE,"cp")) | 1030 |

**Cochrane Library**March 17, 2026

| 1 | [mh "acute coronary syndrome"] OR [mh "acute coronary syndromes"] OR [mh "myocardial infarction"] OR [mh "coronary thrombosis"] OR [mh "coronary occlusion"] | 18472 |
| --- | --- | --- |
| 2 | (“myocardial infarction” OR STEMI OR NSTEMI OR “non-stemi” OR “elevation myocardial infarction” OR OMI OR NOMI OR “occlusion myocardial infarction” OR “occlusion MI” OR “coronary occlusion” OR “coronary artery occlusion”):ti,ab | 36188 |
| 3 | (“hyperacute T wave” OR “hyperacute T waves” OR “symmetrical T wave” OR “symmetrical T waves” OR “prominent T wave” OR “prominent T waves” OR “posterior myocardial infarction” OR “posterior MI” OR “posterior STEMI” OR “posterior wall myocardial infarction” OR “posterior wall MI” OR “posterior wall STEMI” OR posterior NEXT isch?mia OR (ST NEAR/2 depression NEAR/5 (V1 OR V2 OR V3 OR V4)) OR (“anterior ST” NEAR/2 depression) OR Sgarbossa OR (ST NEAR/2 elevation NEAR/4 aVR) OR “aVR sign” OR (“diffuse ST” NEAR/2 depression) OR (“subtle ST” NEAR/2 elevation) OR (“minimal ST” NEAR/2 elevation) OR (“subthreshold ST” NEAR/2 elevation) OR (de NEXT Winter*) OR “upsloping ST” OR “up sloping ST” OR Wellen* OR (“biphasic T” NEXT wave*) OR (“deeply inverted T” NEXT wave*) OR Aslanger* OR “South African flag” OR “terminal QRS distortion” OR “subtle anterior STEMI” OR (“subtle anterior ST” NEAR/2 elevation) OR STEMI NEXT equivalent*) | 584 |
| 4 | #1 OR #2 | 40547 |
| 5 | #4 AND #3 | 186 |
| 6 | (“papillary muscle rupture” OR “free wall rupture” OR “ventricular septal rupture” OR “cardiac rupture” OR “mitral regurgitation” OR hernia OR pancreatitis OR mesenteric OR dissection OR ablation OR covid OR coronavirus OR exercise OR diabet* OR (stress NEXT test*) OR transplant):ti,ab | 319448 |
| 7 | #5 not #6 | 163 |

**Proquest Dissertations & Theses Global**March 17, 2026

| # | Query | Results |
| --- | --- | --- |
| 1 | noft(("myocardial infarction" OR "STEMI" OR "NSTEMI" OR "non-stemi" OR "elevation myocardial infarction" OR "OMI" OR "NOMI" OR "occlusion myocardial infarction" OR "occlusion MI" OR "coronary occlusion" OR "coronary artery occlusion")) | 7432 |
| 2 | ⮑ AND noft(("hyperacute T wave" OR "hyperacute T waves" OR "posterior myocardial infarction" OR "posterior MI" OR "posterior STEMI" OR "posterior wall myocardial infarction" OR "posterior wall MI" OR “posterior wall STEMI” OR “posterior ischemia” or “posterior ischaemia” OR “ST depression” OR “ST segment depression” OR Sgarbossa OR “ST elevation in aVR” OR “ST segment elevation in aVR” OR “subtle ST elevation” OR “subtle ST segment elevation” OR “de Winter” OR “Wellen” OR “Wellen’s” OR “biphasic T wave” OR “biphasic T waves” OR “deeply inverted T wave” OR “deeply inverted T waves” OR “Aslanger” OR “Aslanger’s” OR “South African flag” OR “terminal QRS distortion” OR “subtle anterior STEMI” OR “subtle anterior ST elevation” OR “subtle anterior ST segment elevation” OR “STEMI equivalent”)) | 73 |
| 3 | ⮑ NOT noft((“papillary muscle rupture” OR “free wall rupture” OR “ventricular septal rupture” OR “cardiac rupture” OR “mitral regurgitation” OR hernia OR pancreatitis OR mesenteric OR dissection OR ablation OR covid OR coronavirus OR exercise OR diabetes OR diabetic OR "stress test" OR "stress testing" OR transplant)) | 29 |

**MedRxiv** was searched via Scopus (March 17, 2026), which indexes medRxiv from 2017 onward; however, no medRxiv results were returned. The medRxiv database was hand-searched with simplified terms as a supplementary search but no relevant results were returned.

**Embase (Ovid)**March 17, 2026

| 1 | acute heart infarction/ OR heart infarction size/ OR heart muscle necrosis/ OR heart ventricle infarction/ OR heart left ventricle infarction/ OR heart right ventricle infarction/ OR impending heart infarction/ OR inferior myocardial infarction/ OR non ST segment elevation myocardial infarction/ OR ST segment elevation myocardial infarction/ OR anterior myocardial infarction/ OR coronary occlusion/ OR coronary artery thrombosis/ OR acute coronary syndrome/ OR non st segment elevation acute coronary syndrome/ | 300599 |
| --- | --- | --- |
| 2 | (myocardial infarction or STEMI or NSTEMI or non-stemi or elevation myocardial infarction or OMI or NOMI or occlusion myocardial infarction or occlusion MI or coronary occlusion or coronary artery occlusion).ti,ab. | 78054 |
| 3 | (hyperacute T wave* or symmetrical T wave* or prominent T wave* or posterior myocardial infarction or posterior MI or posterior STEMI or posterior wall myocardial infarction or posterior wall MI or posterior wall STEMI or posterior isch?emia or (ST adj2 depression adj5 (V1 or V2 or V3 or V4)) or (anterior ST adj2 depression) or Sgarbossa or (ST adj2 elevation adj4 aVR) or aVR sign or (diffuse ST adj2 depression) or (subtle ST adj2 elevation) or (minimal ST adj2 elevation) or (subthreshold ST adj2 elevation) or de Winter* or upsloping ST or up sloping ST or Wellen* or biphasic T wave* or deeply inverted T wave* or Aslanger* or South African flag or terminal QRS distortion or subtle anterior STEMI or (subtle anterior ST adj2 elevation) or STEMI equivalent).ti,ab. | 3620 |
| 4 | 1 or 2 | 313890 |
| 5 | 3 and 4 | 1968 |
| 6 | 5 not (papillary muscle rupture or free wall rupture or ventricular septal rupture or cardiac rupture or mitral regurgitation or hernia or pancreatitis or mesenteric or dissection or ablation or covid or coronavirus or exercise or diabet* or stress test* or transplant or stroke? or smoking or ticagrelor or clopidogrel or atorvastatin or sleep or pneumonia or sepsis).ti,ab. | 1334 |
| 7 | 6 not “a case”.ti,ab. | 1122 |

**World Health Organization International Clinical Trials Registry Platform**March 17, 2026

| # | Query | Results |
| --- | --- | --- |
| 1 | "myocardial infarction" OR STEMI OR NSTEMI OR "non-stemi" OR "elevation myocardial infarction" OR OMI OR NOMI OR "occlusion myocardial infarction" OR "occlusion MI" OR "coronary occlusion" OR "coronary artery occlusion" | 6466 |
| 2 | ("hyperacute T wave*" OR "symmetrical T wave*" OR "prominent T wave*" OR "posterior myocardial infarction" OR "posterior MI" OR "posterior STEMI" OR "posterior wall myocardial infarction" OR "posterior wall MI" OR "posterior wall STEMI" OR "posterior ischaemia" OR "posterior ischemia" OR "ST depression" OR "anterior ST depression" OR Sgarbossa OR "ST elevation in aVR" OR "ST segment elevation in aVR" OR "aVR sign" OR "diffuse ST depression" OR "subtle ST elevation" OR "minimal ST elevation" OR "subthreshold ST elevation" OR "de Winter*" OR "upsloping ST" OR "up sloping ST" OR Wellen* OR "biphasic T wave*" OR "deeply inverted T wave*" OR Aslanger* OR "South African flag" OR "terminal QRS distortion" OR "subtle anterior STEMI" OR "subtle anterior ST elevation" OR "STEMI equivalent") | 11  (exported) |
| 3 | ( "myocardial infarction" OR STEMI OR NSTEMI OR "non-stemi" OR "elevation myocardial infarction" OR OMI OR NOMI OR "occlusion myocardial infarction" OR "occlusion MI" OR "coronary occlusion" OR "coronary artery occlusion") AND ("hyperacute T wave*" OR "symmetrical T wave*" OR "prominent T wave*" OR "posterior myocardial infarction" OR "posterior MI" OR "posterior STEMI" OR "posterior wall myocardial infarction" OR "posterior wall MI" OR "posterior wall STEMI" OR "posterior ischaemia" OR "posterior ischemia" OR "ST depression" OR "anterior ST depression" OR Sgarbossa OR "ST elevation in aVR" OR "ST segment elevation in aVR" OR "aVR sign" OR "diffuse ST depression" OR "subtle ST elevation" OR "minimal ST elevation" OR "subthreshold ST elevation" OR "de Winter*" OR "upsloping ST" OR "up sloping ST" OR Wellen* OR "biphasic T wave*" OR "deeply inverted T wave*" OR Aslanger* OR "South African flag" OR "terminal QRS distortion" OR "subtle anterior STEMI" OR "subtle anterior ST elevation" OR "STEMI equivalent") | 0 |

**Open Science Framework**March 17, 2026

| Search | Results |
| --- | --- |
| ("myocardial infarction" \| STEMI \| NSTEMI \| "non-stemi" \| "elevation myocardial infarction" \| OMI \| NOMI \| "occlusion myocardial infarction" \| "occlusion MI" \| "coronary occlusion" \| "coronary artery occlusion") | 389 |
| ("hyperacute T wave*" \| "symmetrical T wave*" \| "prominent T wave*" \| "posterior myocardial infarction" \| "posterior MI" \| "posterior STEMI" \| "posterior wall myocardial infarction" \| "posterior wall MI" \| "posterior wall STEMI" \| "posterior ischaemia" \| "posterior ischemia" \| "ST depression" \| "anterior ST depression" \| Sgarbossa \| "ST elevation in aVR" \| "ST segment elevation in aVR" \| "aVR sign" \| "diffuse ST depression" \| "subtle ST elevation" \| "minimal ST elevation" \| "subthreshold ST elevation" \| "de Winter*" \| "upsloping ST" \| "up sloping ST" \| Wellen* \| "biphasic T wave*" \| "deeply inverted T wave*" \| Aslanger* \| "South African flag" \| "terminal QRS distortion" \| "subtle anterior STEMI" \| "subtle anterior ST elevation" \| "STEMI equivalent") | 62 |
| ("myocardial infarction" \| STEMI \| NSTEMI \| "non-stemi" \| "elevation myocardial infarction" \| OMI \| NOMI \| "occlusion myocardial infarction" \| "occlusion MI" \| "coronary occlusion" \| "coronary artery occlusion") ("hyperacute T wave*" \| "symmetrical T wave*" \| "prominent T wave*" \| "posterior myocardial infarction" \| "posterior MI" \| "posterior STEMI" \| "posterior wall myocardial infarction" \| "posterior wall MI" \| "posterior wall STEMI" \| "posterior ischaemia" \| "posterior ischemia" \| "ST depression" \| "anterior ST depression" \| Sgarbossa \| "ST elevation in aVR" \| "ST segment elevation in aVR" \| "aVR sign" \| "diffuse ST depression" \| "subtle ST elevation" \| "minimal ST elevation" \| "subthreshold ST elevation" \| "de Winter*" \| "upsloping ST" \| "up sloping ST" \| Wellen* \| "biphasic T wave*" \| "deeply inverted T wave*" \| Aslanger* \| "South African flag" \| "terminal QRS distortion" \| "subtle anterior STEMI" \| "subtle anterior ST elevation" \| "STEMI equivalent") | 1 |

**Google Scholar**March 17, 2026

(NSTEMI OR "non-STEMI" OR "elevation myocardial infarction" OR "occlusion myocardial infarction" OR "occlusion MI" OR "coronary occlusion" OR "coronary artery occlusion" OR OMI OR NOMI) AND ("hyperacute T wave" OR "symmetrical T wave" OR "prominent T wave" OR "posterior myocardial infarction" OR "posterior MI" OR "posterior STEMI" OR "posterior wall myocardial infarction" OR "posterior ischaemia" OR "posterior ischemia" OR "anterior ST depression" OR "diffuse ST depression" OR Sgarbossa OR "ST elevation in aVR" OR "ST segment elevation in aVR" OR "aVR sign" OR "subtle ST elevation" OR "minimal ST elevation" OR "subthreshold ST elevation" OR "de Winter" OR "upsloping ST" OR Wellens OR "biphasic T wave" OR "deeply inverted T wave" OR Aslanger OR "South African flag" OR "terminal QRS distortion" OR "subtle anterior STEMI" OR "subtle anterior ST elevation" OR "STEMI equivalent")

200/3,910 results screened

*A single reviewer searched Google Scholar in a logged-out browser session and screened the first 200 results. 10 possibly relevant articles were identified and subsequently imported into Covidence for full screening.*

**Supplemental Searches**

**Medline and Embase (via Ovid)**
April 21, 2026

| # | Query | Results |
| --- | --- | --- |
| 1 | (STEMI or NSTEMI or non-stemi or elevation myocardial infarction or OMI or occlusion myocardial infarction or occlusion MI or coronary occlusion or coronary artery occlusion).ti,ab. | 118836 |
| 2 | (expert interpret* or artificial intelligence or machine learning or blinded).ti,ab. | 834944 |
| 3 | (ECGs or electrocardiograms).ti,ab. | 53330 |
| 4 | 2 and 3 | 4194 |
| 5 | 1 and 4 | 282 |
| 6 | 5 not (heart failure or atrial fibrillation or hypertrophy or stroke or cardiac function).ti,ab. | 229 |

**Scopus**
April 21, 2026

| # | Query | Results |
| --- | --- | --- |
| 1 | TITLE-ABS(STEMI OR NSTEMI OR "non-stemi" OR "elevation myocardial infarction" OR OMI OR "occlusion myocardial infarction" OR "occlusion MI" OR "coronary occlusion" OR "coronary artery occlusion") | 49300 |
| 2 | ⮑ AND TITLE-ABS("expert interpreter” OR “expert interpretation" OR "artificial intelligence" OR "machine learning" OR blinded) | 742 |
| 3 | ⮑ AND TITLE-ABS(ECGs OR electrocardiograms) | 126 |
| 4 | ⮑ AND NOT TITLE-ABS("heart failure" OR "atrial fibrillation" OR hypertrophy OR stroke OR "cardiac function") | 113 |
| 5 | ⮑ AND (LIMIT-TO(DOCTYPE,"ar")) | 100 |

**Supplemental Appendix S2. Sources excluded following full-text review**

| **Year** | **Title** | **Authors** | **Journal** | **DOI** | **Reason** |
| --- | --- | --- | --- | --- | --- |
| **Wrong population (n = 45)** | | | | | |
| 1985 | Electrocardiographic and vectorcardiographic diagnosis of posterior wall myocardial infarction. Significance of the T-wave | Eisenstein, I., et al. | Chest | 10.1378/chest.88.3.409 | Wrong population |
| 1990 | Differentiation between left circumflex and right coronary artery occlusions: Studies on ST-segment deviation during percutaneous transluminal coronary angioplasty | Hiasa, Y., et al. | Clin. Cardiol. | 10.1002/clc.4960131107 | Wrong population |
| 1994 | Simultaneous ST-segment elevation in lead V1 and depression in lead V2. A discordant ECG pattern indicating right ventricular infarction | Mak, K.H., et al. | J. Electrocardiol. | 10.1016/S0022-0736(94)80003-0 | Wrong population |
| 1995 | Evaluation of QRST isointegral maps in detecting posterior myocardial infarction with and without conduction disturbance | Agetsuma, H., et al. | Clin. Cardiol. | 10.1002/clc.4960180207 | Wrong population |
| 1996 | Clinical use of posterior electrocardiographic leads: A prospective electrocardiographic analysis during coronary occlusion | Kulkarni, A.U., et al. | Am. Heart J. | 10.1016/S0002-8703(96)90280-X | Wrong population |
| 1996 | The diagnostic criteria of infarct-related coronary arteries by electrocardiographic ST-segment deviations in the acute stage of myocardial infarction: Prospective study | Suetsuna, R., et al. | Respir. Circ. |  | Wrong population |
| 1997 | Value of leads V7-V9 in diagnosing posterior wall acute myocardial infarction and other causes of tall R-waves in V1-V2 | Casas, R.E., et al. | Am. J. Cardiol. | 10.1016/S0002-9149(97)00404-9 | Wrong population |
| 1999 | Improved detection of posterior myocardial wall ischemia with the 15-lead electrocardiogram | Khaw, K., et al. | Am. Heart J. | 10.1016/S0002-8703(99)70020-7 | Wrong population |
| 2001 | Diagnosis of acute myocardial infarction in angiographically documented occluded infarct vessel : Limitations of ST-segment elevation in standard and extended ECG leads | Schmitt, C., et al. | Chest | 10.1378/chest.120.5.1540 | Wrong population |
| 2001 | New electrocardiographic criteria for posterior wall acute myocardial ischemia validated by a percutaneous transluminal coronary angioplasty model of acute myocardial infarction | Wung, S.-F.; Drew, B.J. | Am. J. Cardiol. | 10.1016/S0002-9149(01)01431-X | Wrong population |
| 2004 | Recognition of left main occlusion in acute coronary syndrome | Chen Y., et al. | Acta Cardiologica Sinica |  | Wrong population |
| 2005 | Significance of lead aVR ST-segment elevation in acute coronary syndrome. | Hengrussamee, Kriengkrai, et al. | Journal of the Medical Association of Thailand = Chotmaihet thangphaet |  | Wrong population |
| 2005 | Differentiating the infarct-related artery on initial electrocardiogram in single or multi-vessel disease in acute inferior myocardial infarction and evaluating involvement of vessels using correspondence analysis | Kürüm, T., et al. | Angiology | 10.1177/000331970505600404 | Wrong population |
| 2005 | Value of lead aVR in the detection of significant left main coronary artery stenosis in acute coronary syndrome | Rostoff P., et al. | Kardiologia Polska | 10.33963/v.kp.81697 | Wrong population |
| 2006 | Unraveling the spectrum of left bundle branch block in acute myocardial infarction: Insights from the assessment of the safety and efficacy of a new thrombolytic (ASSENT 2 and 3) trials | Al-Faleh, H., et al. | Am. Heart J. | 10.1016/j.ahj.2005.02.043 | Wrong population |
| 2006 | Predictive value of lead aVR for lesions in the proximal portion of the left anterior descending coronary artery | Aygül, N., et al. | Turk Kardiyol. Dernegi Ars. |  | Wrong population |
| 2006 | Reciprocal changes in 12-lead electrocardiography can predict left main coronary artery lesion in patients with acute myocardial infarction | Jong, G.-P., et al. | Int. Heart J. | 10.1536/ihj.47.13 | Wrong population |
| 2006 | Prediction of left main coronary artery obstruction by 12-lead electrocardiography: ST-segment deviation in lead V6 greater than or equal to ST-segment deviation in lead V1 | Mahajan N., et al. | Annals of Noninvasive Electrocardiology | 10.1111/j.1542-474X.2006.00090.x | Wrong population |
| 2008 | Acute anterior wall myocardial infarction entailing ST-segment elevation in lead V3R, V1 or aVR: Electrocardiographic and angiographic correlations | Zhong-qun, Z., et al. | J. Electrocardiol. | 10.1016/j.jelectrocard.2007.12.004 | Wrong population |
| 2010 | The ST injury vector: Electrocardiogram-based estimation of location and extent of myocardial ischemia | Andersen, M.P., et al. | J. Electrocardiol. | 10.1016/j.jelectrocard.2009.12.001 | Wrong population |
| 2010 | Early detection of acute posterior myocardial infarction using body surface mapping and SPECT scanning | Neill, J., et al. | Coron. Artery Dis. | 10.1097/MCA.0b013e32833db504 | Wrong population |
| 2012 | Correlation between index electrocardiographic patterns and pre-intervention angiographic findings: Insights from the HORIZONS-AMI trial | Rokos, I.C., et al. | Catheter. Cardiovasc. Interventions | 10.1002/ccd.23262 | Wrong population |
| 2012 | Electrocardiographic differentiation of early repolarization from subtle anterior ST-segment elevation myocardial infarction | Smith, S.W., et al. | Ann. Emerg. Med. | 10.1016/j.annemergmed.2012.02.015 | Wrong population |
| 2013 | The presence of ST-elevation in lead aVR predicts significant left main coronary artery stenosis in cardiogenic shock resulting from myocardial infarction: The Manitoba cardiogenic shock registry | Ducas, R., et al. | Int. J. Cardiol. | 10.1016/j.ijcard.2011.11.003 | Wrong population |
| 2014 | Electrocardiographic characteristics of patients presenting with left main coronary artery ST-elevation myocardial infarction or its equivalent | Chua K., et al. | Global Heart | 10.1016/j.gheart.2014.03.1537 | Wrong population |
| 2014 | A study on the role of ECG changes in localizing the culprit vessel in acute inferior wall myocardial infarction with angiographic correlation in a tertiary care hospital | G., Sylesh Kumar Jain | PQDT - Global |  | Wrong population |
| 2017 | Emergency room evaluation of patients with cardiac complaints and new left bundle branch block: The utility of the Sgarbossa and modified Sgarbossa criteria | Behuria S., et al. | Journal of the American College of Cardiology | 10.1016/S0735-1097%2817%2934659-4 | Wrong population |
| 2017 | The relationship of ST-segment changes in lead aVR with outcomes after myocardial infarction; a cross sectional study | Beyranvand, M.R., et al. | Archives of Academic Emergency Medicine |  | Wrong population |
| 2017 | A new 4-variable formula to differentiate normal variant ST-segment elevation in V2-V4 (early repolarization) from subtle left anterior descending coronary occlusion - adding QRS amplitude of V2 improves the model | Driver, B.E., et al. | J. Electrocardiol. | 10.1016/j.jelectrocard.2017.04.005 | Wrong population |
| 2017 | Position of ST-deviation measurements relative to the J-point: Impact for ischemia detection | Man, S., et al. | J. Electrocardiol. | 10.1016/j.jelectrocard.2016.10.012 | Wrong population |
| 2018 | To determine the diagnostic accuracy of 12-lead ECG for detection of posterior myocardial infarction keeping 15-lead ECG as gold standards | Ahmad, M., et al. | Med. Forum Monthly |  | Wrong population |
| 2018 | A simplified formula discriminating subtle anterior wall myocardial infarction from normal variant ST-segment elevation | Aslanger, E., et al. | Am. J. Cardiol. | 10.1016/j.amjcard.2018.06.053 | Wrong population |
| 2018 | A tale of two formulas: Differentiation of subtle anterior MI from benign ST-segment elevation | Bozbeyoğlu, E., et al. | Ann. Noninvasive Electrocardiol. | 10.1111/anec.12568 | Wrong population |
| 2018 | Electrocardiographic changes in patients with acute myocardial infarction caused by left main trunk occlusion | D'Angelo, C., et al. | J. Cardiovasc. Med. | 10.2459/JCM.0000000000000684 | Wrong population |
| 2019 | Utility of aVR electrocardiogram lead for identifying the culprit lesion in patient with acute coronary syndrome | Patil, V., et al. | J. Datta Meghe Inst. Med. Sci. Univ. | 10.4103/jdmimsu.jdmimsu_106_19 | Wrong population |
| 2020 | Prevalence and association of the Wellens' sign with coronary artery disease in an ethnically diverse urban population | Arshad S., et al. | Journal of Electrocardiology | 10.1016/j.jelectrocard.2020.09.002 | Wrong population |
| 2020 | Clinical characteristics of patients with the de Winter electrocardiogram pattern | Liu, L., et al. | J. Cent. South Univ. Med. Sci. | 10.11817/j.issn.1672-7347.2020.190276 | Wrong population |
| 2021 | Coronary angiographic findings in patients with myocardial infarction with and without ST-segment elevation in lead aVR | Elbehery A.A.E., et al. | Journal of Cardiovascular Disease Research | 10.31838/jcdr.2021.12.07.124 | Wrong population |
| 2023 | Usefulness of the four-variable formula on serial electrocardiograms in detecting subtle anterior myocardial infarction | Sert, E.T., et al. | Am. J. Emerg. Med. | 10.1016/j.ajem.2023.08.032 | Wrong population |
| 2024 | Electrocardiogram patterns to identify left main disease in patients with acute coronary syndrome | Bhandari A., et al. | Journal of the American College of Cardiology | 10.1016/S0735-1097%2824%2903126-7 | Wrong population |
| 2024 | ST-segment elevation in aVR and coronary patterns in acute coronary syndrome (insights from 200 patients) | El-Mhadi S., et al. | Archives of Cardiovascular Diseases | 10.1016/j.acvd.2024.05.007 | Wrong population |
| 2024 | Why do we keep missing left circumflex artery myocardial infarctions? | Geffin, R., et al. | J. Electrocardiol. | 10.1016/j.jelectrocard.2023.12.011 | Wrong population |
| 2025 | Artificial intelligence detection of occlusive myocardial infarction from electrocardiograms interpreted as "normal" by conventional algorithms. | Karim, Shifa R, et al. | Journal of Personalized Medicine | 10.3390/jpm15040130 | Wrong population |
| 2025 | Artificial intelligence-enhanced electrocardiogram detection of acute coronary occlusion in chest pain patients with ST-elevation in lead aVR: A direct comparison to conventional electrocardiogram criteria | Terporten, J., et al. | Eur. Heart J. Acute Cardiovasc. Care | 10.1093/ehjacc/zuaf096 | Wrong population |
| 2026 | Performance of artificial intelligence-powered ECG analysis in suspected ST-segment elevation myocardial infarction. | Sharkey, Scott W, et al. | JACC Advances | 10.1016/j.jacadv.2026.102671 | Wrong population |
| **Wrong concept (n = 39)** | | | | | |
| 1987 | Electrocardiographic evolution of posterior acute myocardial infarction: Importance of early precordial ST-segment depression | Boden, W.E., et al. | Am. J. Cardiol. | 10.1016/0002-9149(87)91091-5 | Wrong concept |
| 1988 | Unstable angina: ST-segment depression with positive versus negative T-wave deflections--clinical course, ECG evolution, and angiographic correlation | Sclarovsky, S., et al. | Am Heart J | 10.1016/0002-8703(88)90143-3 | Wrong concept |
| 1989 | Classification of acute myocardial ischemia by electrocardiography | Sclarovsky, S., et al. | Harefuah |  | Wrong concept |
| 1995 | Electrocardiographic characteristics of patients with left circumflex- related myocardial infarction in the acute phase without tented T-waves or definite ST-elevation | Kanemoto, N., et al. | J. Cardiol. |  | Wrong concept |
| 1995 | Electrocardiography and coronary angiographic findings in patients with posterior wall myocardial infarction | Mar, G.-Y., et al. | Acta Cardiol. Sin. |  | Wrong concept |
| 1999 | Importance of posterior chest leads in patients with suspected myocardial infarction, but nondiagnostic, routine 12-lead electrocardiogram | Agarwal, J.B., et al. | Am. J. Cardiol. | 10.1016/S0002-9149(98)00861-3 | Wrong concept |
| 1999 | Prevalence and outcome of ST-segment elevation in posterior electrocardiographic leads during acute myocardial infarction | Oraii, S., et al. | J. Electrocardiol. | 10.1016/S0022-0736(99)90110-3 | Wrong concept |
| 2000 | The correlation between presenting ST-segment depression and the final size of acute myocardial infarcts in patients with acute coronary syndromes | Asfour, W., et al. | J. Electrocardiol. | 10.1054/jelc.2000.20338 | Wrong concept |
| 2007 | Detection of proximal coronary occlusion in acute coronary syndrome: A feasibility study using computerized electrocardiographic analysis | Eskola, M.J., et al. | Ann. Noninvasive Electrocardiol. | 10.1111/j.1542-474X.2007.00179.x | Wrong concept |
| 2007 | Acute coronary syndrome- ECG-changes without ST-elevation | Forselv, G.C.; Vik-Mo, H. | Tidsskr. Nor. Laegeforen. |  | Wrong concept |
| 2011 | The epidemiological and prognostic importance of the aVR lead among patients with and without ST-segment elevation | Coşkun, A., et al. | Turk. J. Thorac. Cardiovasc. Surg. | 10.5606/tgkdc.dergisi.2011.089 | Wrong concept |
| 2011 | Utility of left bundle branch block as a diagnostic criterion for acute myocardial infarction | Jain, S., et al. | Am. J. Cardiol. | 10.1016/j.amjcard.2010.12.007 | Wrong concept |
| 2011 | Short and long-term prognostic significance of ST-segment elevation in lead aVR in patients with non-ST-segment elevation acute coronary syndrome | Taglieri N., et al. | Journal of the American College of Cardiology | 10.1016/S0735-1097%2811%2961063-2 | Wrong concept |
| 2012 | Angiocardiographic findings in patients with biphasic T-wave inversion in precordial leads | Akhtar P., et al. | Journal of the Pakistan Medical Association |  | Wrong concept |
| 2013 | Can Wellens' sign be used to predict significant proximal left anterior descending artery lesion? | Alderwish E., et al. | Circulation: Cardiovascular Quality and Outcomes | 10.1161/circoutcomes.6.suppl_1.a51 | Wrong concept |
| 2013 | Diffuse ST-depression with ST-elevation in aVR: Is this pattern specific for global ischemia due to left main coronary artery disease? | Knotts R.J., et al. | Journal of Electrocardiology | 10.1016/j.jelectrocard.2012.12.016 | Wrong concept |
| 2014 | Diagnosis of acute myocardial infarction in patients with complete left bundle branch block: Prospective evaluation of the modified Sgarbossa criteria (Smith criteria) | Jaeger C., et al. | European Heart Journal: Acute Cardiovascular Care | 10.1177/2048872614549721 | Wrong concept |
| 2015 | Is there a clinical difference in patients with acute coronary syndrome displaying Wellens type 1 versus type 2 patterns on ECG? | Kim J., et al. | Heart Lung and Circulation | 10.1016/j.hlc.2015.06.095 | Wrong concept |
| 2015 | Prevalence and clinical significance of up-sloping ST-segment depression in patients with non-ST-segment elevation myocardial infarction | Misumida, N., et al. | Cardiology Research | 10.14740/cr422w | Wrong concept |
| 2016 | Predictive value of ST-segment elevation in lead aVR for left main and/or three-vessel disease in non-ST-segment elevation myocardial infarction | Misumida, N., et al. | Ann. Noninvasive Electrocardiol. | 10.1111/anec.12272 | Wrong concept |
| 2017 | Chicken or the egg: ST-elevation in lead aVR or SYNTAX score | Cerit, L. | Cardiovas. J. Afri. | 10.5830/CVJA-2016-062 | Wrong concept |
| 2017 | Recognition and management of posterior myocardial infarction: A retrospective cohort study | White, L.D., et al. | Br. J. Cardiol. | 10.5837/bjc.2017.011 | Wrong concept |
| 2018 | New or presumed new left bundle branch block in patients with suspected ST-elevation myocardial infarction. | Pera, Vijaya K, et al. | European heart journal. Acute cardiovascular care | 10.1177/2048872617691508 | Wrong concept |
| 2019 | Prevalence of junctional ST-depression with tall symmetrical T-waves in a pre-hospital field triage system for STEMI patients | de Winter, R.W., et al. | J. Electrocardiol. | 10.1016/j.jelectrocard.2018.10.092 | Wrong concept |
| 2019 | Clinical data analysis of de Winter syndrome | Diao, F., et al. | J. Intervent. Radiol. | 10.3969/j.issn.1008-794X.2019.04.003 | Wrong concept |
| 2020 | New electrocardiographic algorithm for the diagnosis of acute myocardial infarction in patients with left bundle branch block | Di Marco, A., et al. | J Am Heart Assoc | 10.1161/JAHA.119.015573 | Wrong concept |
| 2020 | Electrocardiographic identification of the culprit coronary artery in acute non-ST-elevation myocardial infarction: Predictive value of N-wave and T-wave precordial instability | Rostoff, P., et al. | Coron. Artery Dis. | 10.1097/MCA.0000000000000918 | Wrong concept |
| 2021 | Mid term outcomes in Wellens syndrome | Avram, A., et al. | Rom. Med. J. | 10.37897/RMJ.2021.2.15 | Wrong concept |
| 2021 | The added value of ST-elevation in lead aVR to clinical TIMI score in predicting the angiographic severity of coronary artery disease in patients with non-ST-elevation myocardial infarction | Elfahham A.F., et al. | QJM | 10.1093/qjmed/hcab090.001 | Wrong concept |
| 2021 | Association of ST-elevation in lead aVR with left main stem and triple vessel diseases in patients with non-ST-elevation myocardial infarction | Usman, H.T., et al. | Pakistan Heart J. | 10.47144/phj.v54i4.2128 | Wrong concept |
| 2022 | Association of aVR ST-elevation with outcomes in patients with acute coronary syndrome | Cordovez R.A. | European Heart Journal | 10.1093/eurheartj/ehab849.083 | Wrong concept |
| 2022 | STEMI equivalents and their incidence during EMS transport | Palladino, N., et al. | Prehosp. Emerg. Care | 10.1080/10903127.2020.1863533 | Wrong concept |
| 2023 | Incidence, clinical presentation and predictors of left main coronary artery disease in high-risk patients with a first episode of non-ST-elevation acute coronary syndrome | Chattopadhyay S., et al. | European Journal of Cardiovascular Medicine |  | Wrong concept |
| 2023 | Diagnostic test accuracy of life-threatening electrocardiographic findings (ST-elevation myocardial infarction equivalents) for acute coronary syndrome after out-of-hospital cardiac arrest without ST-segment elevation | Yoshimura, S., et al. | Resuscitation | 10.1016/j.resuscitation.2023.109700 | Wrong concept |
| 2024 | Early predictors of severe left main and/or three-vessel disease in patients with non-ST-segment elevation myocardial infarction: A dual-center retrospective study | Huang, B., et al. | Ann. Noninvasive Electrocardiol. | 10.1111/anec.13120 | Wrong concept |
| 2024 | Characteristics of inferior myocardial infarction with a special electrocardiographic pattern (Aslanger) in metabolic syndrome | Tretyakov, A.Yu., et al. | Kardiologiya | 10.18087/cardio.2024.2.n2532 | Wrong concept |
| 2025 | Diagnostic accuracy of Wellens’ syndrome in predicting significant proximal left anterior descending artery stenosis among patients with acute coronary syndrome | Mujtaba, K.M., et al. | Pakistan Heart J. | 10.47144/phj.v58is3.3308 | Wrong concept |
| 2025 | Prevalence of occlusive myocardial infarction in patients diagnosed with non-ST-elevation myocardial infarction at a single private facility in Cape Town, South Africa | Schoeman, E., et al. | Cardiovascular Journal of Africa | 10.5830/CVJA-2025-074 | Wrong concept |
| 2026 | Real-time integration of an AI-based ECG interpretation system in the emergency department: A pragmatic alternating-day study of diagnostic performance and clinical process metrics | Choi, M.S., et al. | Healthcare (Basel) | 10.3390/healthcare14070968 | Wrong concept |
| **Wrong study design (n = 44)** | | | | | |
| 1991 | Isolated ST-segment depression in leads V2 to V4: An early electrocardiographic sign of posterior myocardial infarction | Roul, G., et al. | Arch. Mal. Coeur Vaiss. |  | Wrong study design |
| 2010 | For diagnosis of acute anterior myocardial infarction due to left anterior descending artery occlusion in left bundle branch block, high ST/S ratio is more accurate than convex ST-segment morphology | Dodd K., et al. | Academic Emergency Medicine | 10.1111/j.1553-2712.2010.00743.x | Wrong study design |
| 2012 | The value of ST-segment elevation in lead aVR for predicting left main coronary artery lesion in patients suspected of acute coronary syndrome | Nough H., et al. | Romanian journal of internal medicine = Revue roumaine de medecine interne |  | Wrong study design |
| 2013 | Modifying the Sgarbossa criteria to diagnose ST-elevation myocardial infarction in the presence of left bundle branch block: A comparison of the Smith ST/QRS ratio rule to the Selvester 10% rule | Dodd K.W.; Smith S.W. | Academic Emergency Medicine | 10.1111/acem.12115 | Wrong study design |
| 2013 | Circumflex artery-related ST-elevation myocardial infarction is associated with an increased delay in primary PCI: Data from the Austrian acute PCI registry | Dorler J., et al. | Wiener Klinische Wochenschrift | 10.1007/s00508-013-0372-9 | Wrong study design |
| 2013 | The role of electrocardiogram for left main coronary artery occlusion detection in patients with acute coronary syndrome | Sciarra F., et al. | Cardiology (Switzerland) | 10.1159/000355890 | Wrong study design |
| 2014 | For diagnosis of acute coronary occlusion in left bundle branch block, ST/QRS ratio is more accurate than T-wave concordance | Dodd K.W., et al. | Annals of Emergency Medicine | 10.1016/j.annemergmed.2014.07.072 | Wrong study design |
| 2014 | Electrocardiographic diagnosis of acute myocardial infarction in paced rhythms: Utility of the Sgarbossa criteria | Fordyce C.B., et al. | Canadian Journal of Cardiology |  | Wrong study design |
| 2014 | Suspected left bundle branch block equivalent STEMI: Analysis in a primary PCI programme | O'Brien J., et al. | European Heart Journal | 10.1093/eurheartj/ehu325 | Wrong study design |
| 2015 | ST-segment changes in left bundle branch block with acute coronary occlusion: Concordance has high specificity while proportionally excessive discordance has high sensitivity | Dodd K.W.; Smith S.W. | Academic Emergency Medicine | 10.1111/acem.12644 | Wrong study design |
| 2015 | T-wave changes aid in the electrocardiographic diagnosis of acute coronary occlusion in left bundle branch block | Elm K.D., et al. | Annals of Emergency Medicine | 10.1016/j.annemergmed.2015.07.146 | Wrong study design |
| 2015 | ECG evaluation in patients with pacemaker and suspected ACS: Which score to apply? | Freitas P., et al. | European Heart Journal: Acute Cardiovascular Care | 10.1177/2048872615599730 | Wrong study design |
| 2015 | Identification of acute coronary syndrome in bi-ventricular paced electrocardiograms | Singh J., et al. | Catheterization and Cardiovascular Interventions | 10.1002/ccd.25910 | Wrong study design |
| 2015 | Post-resuscitation ECG for selection of patients for immediate coronary angiography in out-of-hospital cardiac arrest. | Staer-Jensen, Henrik, et al. | Circulation: Cardiovascular Interventions | 10.1161/CIRCINTERVENTIONS.115.002784 | Wrong study design |
| 2016 | S-wave voltage in V1 to V3 is lower in left bundle branch block patients with acute left anterior descending artery occlusion | Dodd K.W.; Smith S.W. | Academic Emergency Medicine | 10.1111/acem.12974 | Wrong study design |
| 2017 | New ECG criteria for the diagnosis of acute myocardial infarction in the presence of left bundle branch block: A multicenter study among patients referred for primary PCI | Di Marco A., et al. | European Heart Journal | 10.1093/eurheartj/ehx502.P4658 | Wrong study design |
| 2017 | Electrocardiographic parameters, peak of cardiac markers and diagnosis delay in patients with acute left circumflex occlusion | Perez-Diaz P., et al. | Journal of the American College of Cardiology | 10.1016/j.jacc.2017.09.876 | Wrong study design |
| 2017 | Correlation between electrocardiographic changes and coronary findings in patients with acute myocardial infarction and single-vessel disease. | Sanaani, Abdallah, et al. | Annals of Translational Medicine | 10.21037/atm.2017.06.33 | Wrong study design |
| 2018 | The Smith-Modified Sgarbossa criteria accurately diagnose acute coronary occlusion in emergency department patients with ventricular paced rhythm | Dodd K.W., et al. | Academic Emergency Medicine | 10.1111/acem.13424 | Wrong study design |
| 2018 | T-wave changes in patients with ventricular-paced rhythm and acute coronary occlusion | Dodd K.W., et al. | Academic Emergency Medicine | 10.1111/acem.13424 | Wrong study design |
| 2020 | ST-segment elevation myocardial infarction (STEMI) due to left circumflex coronary artery stenosis: Electrocardiographic parameters, infarct size, reperfusion delay and prognosis | Diaz P.P.P., et al. | European Journal of Preventive Cardiology | 10.1177/2047487320935268 | Wrong study design |
| 2020 | The de Winter ECG pattern: Distribution and morphology of ST-depression. | Zhan, Zhong-Qun, et al. | Annals of noninvasive electrocardiology : the official journal of the International Society for Holter and Noninvasive Electrocardiology, Inc | 10.1111/anec.12783 | Wrong study design |
| 2021 | Acute circumflex coronary artery occlusion; dilemma in diagnosis and management | Komatsu J., et al. | European Heart Journal | 10.1093/eurheartj/ehab724.1476 | Wrong study design |
| 2021 | Comparison of the ST-elevation myocardial infarction (STEMI) vs. NSTEMI and occlusion MI (OMI) vs. NOMI paradigms of acute MI | Meyers, H. P., et al. | J Emerg Med | 10.1016/j.jemermed.2020.10.026 | Wrong study design |
| 2021 | Accuracy of OMI ECG findings versus STEMI criteria for diagnosis of acute coronary occlusion myocardial infarction | Meyers, H., et al. | Int J Cardiol Heart Vasc | 10.1016/j.ijcha.2021.100767 | Wrong study design |
| 2022 | Differential electrocardiographic changes related to the culprit coronary artery in patients with acute myocardial infarction and left bundle branch block | Sole Gonzalez E., et al. | European Heart Journal | 10.1093/eurheartj/ehac544.1425 | Wrong study design |
| 2023 | Machine learning for ECG diagnosis and risk stratification of occlusion myocardial infarction | Al-Zaiti, S.S., et al. | Nat. Med. | 10.1038/s41591-023-02396-3 | Wrong study design |
| 2024 | International evaluation of an artificial intelligence-powered electrocardiogram model detecting acute coronary occlusion myocardial infarction. | Herman, Robert, et al. | European Heart Journal Digital Health | 10.1093/ehjdh/ztad074 | Wrong study design |
| 2024 | OMI/NOMI: Time for a new classification of acute myocardial infarction | Kola, M., et al. | J Clin Med | 10.3390/jcm13175201 | Wrong study design |
| 2024 | TCT-194 evaluating artificial intelligence prediction of occlusive myocardial infarction from 12-lead ECGs after resuscitated out of hospital cardiac arrest | Sajjad U., et al. | Journal of the American College of Cardiology | 10.1016/j.jacc.2024.09.230 | Wrong study design |
| 2024 | Testing the accuracy of automatic ECG-annotation for suspected acute coronary syndrome | Sams L.E., et al. | European Heart Journal | 10.1093/eurheartj/ehae666.1360 | Wrong study design |
| 2025 | TCT-1204 retrospective assessment of an artificial intelligence (AI)-based ECG model for detecting acute coronary occlusion (ACO) in a diverse community hospital setting | Aziz I.N., et al. | Journal of the American College of Cardiology | 10.1016/j.jacc.2025.09.1427 | Wrong study design |
| 2025 | Novel artificial intelligence model using electrocardiogram for detecting acute myocardial infarction needing revascularization. | Cho, Kyung Hoon, et al. | European Heart Journal Digital Health | 10.1093/ehjdh/ztaf049 | Wrong study design |
| 2025 | Derivation of an artificial intelligence-based electrocardiographic model for the detection of acute coronary occlusive myocardial infarction. | Diaz-Herrera, Braiana A, et al. | Archivos de Cardiologia de Mexico | 10.24875/ACM.24000195 | Wrong study design |
| 2025 | Validation of an artificial intelligence model for occlusion myocardial infarction identification: Initial findings from a Portuguese cohort | Grine M., et al. | European Heart Journal | 10.1093/eurheartj/ehaf784.4458 | Wrong study design |
| 2025 | External validation of the ECG-SMART model: A machine learning algorithm for ECG diagnosis of occlusion myocardial infarction | Lopez Ayala P., et al. | European Heart Journal | 10.1093/eurheartj/ehaf784.1994 | Wrong study design |
| 2025 | Beyond STEMI: high-risk ECG patterns as predictors of occlusive myocardial infarction in out-of-hospital cardiac arrest patients | Silwanis, C., et al. | Resuscitation | 10.1016/j.resuscitation.2025.110763 | Wrong study design |
| 2026 | AI-enhanced recognition of occlusions in acute coronary syndrome (AERO-ACS): A retrospective study. | Choi, James W H, et al. | Coronary Artery Disease | 10.1097/MCA.0000000000001555 | Wrong study design |
| 2026 | Can an artificial intelligence electrocardiogram algorithm improve diagnostic accuracy for acute coronary occlusion in the difficult subset of canceled catheterization lab activations?. | Friedman, Brandon S, et al. | Journal of Electrocardiology | 10.1016/j.jelectrocard.2026.154255 | Wrong study design |
| 2026 | Detecting occlusion myocardial infarction with an AI-powered ECG model: A retrospective cohort study | Hellerman, Mark B., et al. | Journal of Personalized Medicine | 10.3390/jpm16040174 | Wrong study design |
| 2026 | AI-enabled ECG analysis improves diagnostic accuracy and reduces false STEMI activations | Herman, R., et al. | JACC Cardiovasc. Interventions | 10.1016/j.jcin.2025.10.018 | Wrong study design |
| 2026 | A new and modified northern occlusion myocardial infarction pattern | Ni H., et al. | Journal of electrocardiology | 10.1016/j.jelectrocard.2026.154212 | Wrong study design |
| 2026 | Predicting occlusion myocardial infarctions in the emergency department using artificial intelligence | Nyström, A., et al. | JACEP Open | 10.1016/j.acepjo.2025.100299 | Wrong study design |
| 2026 | Artificial intelligence versus human expertise: ECG-based detection of occlusive myocardial infarction after cardiac arrest | Silwanis, C., et al. | Resuscitation | 10.1016/j.resuscitation.2025.110905 | Wrong study design |
| **Wrong context (n = 1)** | | | | | |
| 2009 | Usefulness of three posterior chest leads for the detection of posterior wall acute myocardial infarction | Aqel, R.A., et al. | Am. J. Cardiol. | 10.1016/j.amjcard.2008.09.008 | Wrong context |
| **Full text unavailable (n = 7)** | | | | | |
| 2013 | Clinical profile and angiographic correlation in Wellens' syndrome | Bathri Narayanan R., et al. | Indian Heart Journal | org/10.1016/j.ihj.2013.10.018 | Full text unavailable |
| 2021 | STEMI equivalent ECG changes in NSTEMI | Borse A.G., et al. | Indian Heart Journal | 10.1016/j.ihj.2021.11.122 | Full text unavailable |
| 2021 | ST-elevation in aVR lead: Risk factors and clinical importance | Hossein S., et al. | Acta Medica Mediterranea | 10.19193/0393-6384_2021_3_242 | Full text unavailable |
| 2021 | Electrocardiogram diagnosis of acute myocardial infarction with complete left bundle branch block | Zhong, T., et al. | J. Clin. Cardiol. | 10.13201/j.issn.1001-1439.2021.09.011 | Full text unavailable |
| 2024 | Retrospective analysis of acute coronary syndrome (ACS) presenting with Wellens' pattern on electrocardiography (ECG) | Tiwari D.K.; Belle C.U. | Heart | 10.1136/heartjnl-2024-BCS.70 | Full text unavailable |
| 2025 | Spotting a novel pattern for myocardial infarction | Darak A., et al. | European Heart Journal | 10.1093/eurheartj/ehaf784.1727 | Full text unavailable |
| 2025 | ECG patterns in patients presenting with NSTEMI having occlusion myocardial infarction, a retrospective analysis | Thomas V.M., et al. | Indian Heart Journal | 10.1016/j.ihj.2025.11.201 | Full text unavailable |

**Supplemental Appendix S3. Characteristics of included studies**

| **Citation** | **Study design** | **Context** | **ECG pattern(s) examined** | **Reference standard** | **Data sources (n)** |
| --- | --- | --- | --- | --- | --- |
| Alireza et al. (2025)^1^ | Prospective cohort | Inpatient / hospital | de Winter | Composite | 1 |
| Aslanger et al. (2020a)^2^ | Retrospective cohort | Emergency department | Hyperacute T waves or de Winter pattern Subtle anterior STE Minor STE with reciprocal ST-depression Nonconsecutive STE | Composite | 4 |
| Aslanger et al. (2020b)^3^ | Retrospective case-control | Inpatient/hospital | Aslanger pattern | Composite | 1 |
| Bar-Yishay et al. (2010)^4^ | Retrospective cohort | Inpatient / CCU | Isolated inferior STD | Angiographic | 1 |
| Bischof et al. (2016)^5^ | Retrospective cohort | Emergency department | Any degree of inferior STE + STD in aVL | Angiographic | 1 |
| Carvalho et al. (2026)^6^ | Retrospective cohort | Inpatient/hospital | Hyperacute T waves Subtle STE | Composite | 2 |
| Chelakkat M. et al. (2025)^7^ | Prospective cross-sectional | Inpatient / hospital | Wellens | Angiographic | 1 |
| de la Torre Fonseca et al. (2023)^8^ | Prospective cohort | Inpatient / hospital | Wellens | Angiographic | 1 |
| de Zwaan et al. (1989)^9^ | Prospective cohort | Inpatient/hospital | Wellens | Angiographic | 1 |
| Di Marco et al. (2017)^10^ | Retrospective cohort | ED, pPCI network | Sgarbossa Smith-Modified Sgarbossa | Composite | 2 |
| Dodd et al. (2016)^11^ | Retrospective cohort | Emergency department | Smith-Modified Sgarbossa Criteria (with 0.5 mm cutoff) | Composite | 1 |
| Dodd et al. (2021)^12^ | Retrospective case-control | Emergency department | Unweighted Sgarbossa Smith-Modified Sgarbossa Smith-Modified Sgarbossa with extended rule 2 | Composite | 3 |
| Freitas et al. (2016)^13^ | Prospective registry | Inpatient/hospital | Weighted Sgarbossa Smith-Modified Sgarbossa Selvester Criteria | Composite | 3 |
| Fujii T. et al. (2024)^14^ | Retrospective observational | Inpatient / hospital | de Winter Wellens | Angiographic | 2 |
| Gołąbek et al. (2023)^15^ | Retrospective cohort | Inpatient / hospital | Wellens STE-aVR | Angiographic | 2 |
| Goss et al. (2025)^16^ | Retrospective cohort | Emergency department | Precordial Swirl 1, 2, 3 | Composite | 3 |
| Harhash et al. (2019)^17^ | Retrospective cohort | Emergency department | STE-aVR with diffuse STD | Angiographic | 1 |
| Horie et al. (2022)^18^ | Retrospective cohort | Emergency department | Synthesized posterior STE STD V1-V3 | Angiographic | 2 |
| Jang et al. (2014)^19^ | Retrospective cohort | Inpatient / cath lab | Minimal STE | Angiographic | 1 |
| Kobayashi et al. (2019)^20^ | Retrospective cohort | Inpatient / hospital | Wellens | Angiographic | 1 |
| Komatsu et al. (2022)^21^ | Retrospective cohort | Inpatient/hospital | Isolated anterior STD | Angiographic | 1 |
| Lai et al. (2020)^22^ | Retrospective cohort | Emergency department | Smith-Modified Sgarbossa | Composite | 1 |
| Lindow et al. (2024)^23^ | Prospective registry | Emergency department | Sgarbossa Smith-Modified Sgarbossa Barcelona Criteria Selvester Criteria | Angiographic | 4 |
| Liu et al. (2022)^24^ | Retrospective cohort | Inpatient / cath lab | STE-aVR STE in aVR + aVL | Angiographic | 2 |
| McMahon et al. (2013)^25^ | Retrospective cohort | Mixed (pre-hospital / ED) | Sgarbossa Criteria (individual components) | Angiographic | 1 |
| Meng et al. (2022)^26^ | Retrospective cohort | Inpatient / hospital | Wellens Isolated STD in V1-V4 Prominent R in V1 | Angiographic | 3 |
| Meyers et al. (2015)^27^ | Retrospective case-control | Emergency department | Sgarbossa Smith-Modified Sgarbossa | Composite | 2 |
| Meyers et al. (2021)^28^ | Retrospective case-control | Emergency department | Suspected primary ischemic STDmaxV1-4 | Composite | 1 |
| Meyers et al. (2025a)^29^ | Retrospective cohort | Emergency department | Hyperacute T Waves | Angiographic / Composite | 2 |
| Meyers et al. (2025b)^30^ | Retrospective cohort (substudy) | Emergency department | Hyperacute T waves (including de Winter) Terminal QRS distortion Reciprocal STD and/or T wave inversion Subtle STE not meeting STEMI criteria Any degree of inferior STE + STD in aVL | Angiographic | 5 |
| Pride et al. (2010)^31^ | Retrospective substudy (RCT) | Mixed / multicentre (TRITON-TIMI 38) | Isolated STD V1-V4 | Composite | 1 |
| Schmitt et al. (2001)^32^ | Retrospective cohort | Inpatient / hospital | Isolated STE V7-V9 | Angiographic | 1 |
| Smith et al. (2012)^33^ | Retrospective case-control | Emergency department | Weighted Sgarbossa Unweighted Sgarbossa Smith-Modified Sgarbossa | Composite | 3 |
| Tang et al. (2024)^34^ | Retrospective matched cohort | Inpatient / hospital | de Winter | Angiographic | 1 |
| Verouden et al. (2009)^35^ | Retrospective cohort | Mixed (pre-hospital / cath lab) | de Winter | Angiographic | 1 |
| Vives-Borrás et al. (2017)^36^ | Retrospective cohort | Inpatient/hospital | Isolated anterior STD | Angiographic | 1 |
| Wall et al. (2018)^37^ | Retrospective cohort | Cath lab | de Winter STD V1-V4 | Angiographic | 2 |
| Wei et al. (2025)^38^ | Retrospective cohort | Inpatient/hospital | Prominent R in V1 Isolated anterior STD de Winter STD in I, aVL | Angiographic | 4 |
| Wen et al. (2021)^39^ | Retrospective cohort | Inpatient / hospital, ECGs obtained in ED and prehospital. | de Winter | Angiographic | 1 |
| Wiśniewski et al. (2019)^40^ | Retrospective cohort | Inpatient / hospital | STE-aVR de Winter STD V1-V4 STD I, aVL, V6 | Angiographic | 4 |
| Xu et al. (2018)^41^ | Retrospective cohort | ED / Inpatient / hospital | de Winter | Angiographic | 1 |
| Yamamoto et al. (2019)^42^ | Retrospective cohort | Emergency department / post-cardiac arrest | Isolated STE aVR (post-ROSC, immediate) Isolated STE aVR (post-ROSC, follow-up) | Angiographic | 2 |

## Appendix S3 Reference List

1. Alireza M, Nader A, Alireza F, Babak B, Bahare G. Traces of cardioprotection behind the uncertainty of the de Winter pattern. J Electrocardiol 2025;92:154056. https://doi.org/10.1016/j.jelectrocard.2025.154056

2. Aslanger E, Yıldırımtürk Ö, Şimşek B, Bozbeyoğlu E, Şimşek M, Yücel Karabay C, et al. Diagnostic accuracy of electrocardiogram for acute coronary occlusion resulting in myocardial infarction (DIFOCCULT study). Int J Cardiol Heart Vasc 2020;30:100603. https://doi.org/10.1016/j.ijcha.2020.100603

3. Aslanger E, Yıldırımtürk Ö, Şimşek B, Sungur A, Türer Cabbar A, Bozbeyoğlu E, et al. A new electrocardiographic pattern indicating inferior myocardial infarction. J Electrocardiol 2020;61:41-46. https://doi.org/10.1016/j.jelectrocard.2020.04.008

4. Bar-Yishay I, Gilutz H, Cafri C, Ilia R, Zahger D. Isolated inferior wall ST segment depression as an early sign of acute anterior wall myocardial infarction. Acute Card Care 2010;12(4):119-123. https://doi.org/10.3109/17482941.2010.528429

5. Bischof J, Worrall C, Thompson P, Marti D, Smith S. ST depression in lead aVL differentiates inferior ST-elevation myocardial infarction from pericarditis. Am J Emerg Med 2016;34(2):149-154. https://doi.org/10.1016/j.ajem.2015.09.035

6. Carvalho PEP, Belzer W, Pollmann DL, Helseth HC, Traverse JH, Herman R, et al. AI-enhanced electrocardiogram for detection of occlusive myocardial infarction in high-risk non-ST-segment elevation acute coronary syndrome. JACC Adv 2026;5(4):102663. https://doi.org/10.1016/j.jacadv.2026.102663

7. Chelakkat M, Karunadas CP, Bijilesh U, Mathew C. Significance of Wellens pattern in electrocardiogram revisited - electrocardiogram and angiographic correlation. Journal of Indian College of Cardiology 2025;15(1):13-17. https://doi.org/10.4103/jicc.jicc_13_24

8. de la Torre Fonseca L, Alarcón Cedeño R, Jiménez Díaz V, Wang L, Loor Cedeño F, Juan-Salvadores P. Wellens syndrome as an independent predictor of in-hospital cardiovascular complications. Acta Cardiol 2023;78(6):680-686. https://doi.org/10.1080/00015385.2022.2093797

9. de Zwaan C, Bär FW, Janssen JHA, Cheriex EC, Dassen WRM, Brugada P, et al. Angiographic and clinical characteristics of patients with unstable angina showing an ECG pattern indicating critical narrowing of the proximal LAD coronary artery. Am Heart J 1989;117(3):657-665. https://doi.org/10.1016/0002-8703(89)90742-4

10. Di Marco A, Anguera I, Rodríguez M, Sionis A, Bayes-Genis A, Rodríguez J, et al. Assessment of Smith algorithms for the diagnosis of acute myocardial infarction in the presence of left bundle branch block. Rev Esp Cardiol 2017;70(7):559-566. https://doi.org/10.1016/j.recesp.2016.11.010

11. Dodd K, Elm K, Smith S. Comparison of the QRS complex, ST segment, and T wave among patients with left bundle branch block with and without acute myocardial infarction. J Emerg Med 2016;51(1):1-8. https://doi.org/10.1016/j.jemermed.2016.02.029

12. Dodd K, Zvosec D, Hart M, Glass G, Bannister L, Body R, et al. Electrocardiographic diagnosis of acute coronary occlusion myocardial infarction in ventricular paced rhythm using the modified Sgarbossa criteria. Ann Emerg Med 2021;78(4):517-529. https://doi.org/10.1016/j.annemergmed.2021.03.036

13. Freitas P, Santos MB, Faria M, Rodrigues G, Vale N, Teles RC, et al. ECG evaluation in patients with pacemaker and suspected acute coronary syndrome: which score should we apply? J Electrocardiol 2016;49(5):744-748. https://doi.org/10.1016/j.jelectrocard.2016.06.012

14. Fujii T, Ikari Y. Clinical outcomes in acute coronary syndrome after presentation of unique electrocardiographic findings. J Electrocardiol 2024;85:31-36. https://doi.org/10.1016/j.jelectrocard.2024.05.100

15. Gołąbek N, Jakubowski W, Król S, Kozioł M, Niewiara Ł, Kleczyński P, et al. ECG patterns suggestive of high-risk coronary anatomy in non-ST-segment elevation acute coronary syndrome - an analysis of real-world patients. Postepy Kardiol Interwencyjnej 2023;19(4):326-332. https://doi.org/10.5114/aic.2023.132161

16. Goss L, Meyers H, Friedman B, Bracey A, Smith S. Precordial swirl sign: a new ECG pattern of left anterior descending artery occlusion myocardial infarction. J Electrocardiol 2025;91:153931. https://doi.org/10.1016/j.jelectrocard.2025.153931

17. Harhash AA, Huang JJ, Reddy S, Natarajan B, Balakrishnan M, Shetty R, et al. aVR ST segment elevation: acute STEMI or not? Incidence of an acute coronary occlusion. Am J Med 2019;132(5):622-630. https://doi.org/10.1016/j.amjmed.2018.12.021

18. Horie T, Hamaya R, Sugiyama T, Hirano H, Hoshino M, Kanaji Y, et al. Synthesized 18-lead electrocardiogram in diagnosing posterior STEMI-equivalent acute coronary syndrome in patients with NSTEMI. Cardiol Res Pract 2022;2022:9582174. https://doi.org/10.1155/2022/9582174

19. Jang S, Bae M, Kim J, Park S, Lee J, Yang D, et al. Predictors and clinical implications of minimal ST-segment elevation in patients with ST-segment elevation myocardial infarction. Cardiology 2014;128(3):273-281. https://doi.org/10.1159/000362438

20. Kobayashi A, Misumida N, Aoi S, Kanei Y. Prevalence and clinical implication of Wellens’ sign in patients with non-ST-segment elevation myocardial infarction. Cardiol Res 2019;10(3):135-141. https://doi.org/10.14740/CR856

21. Komatsu J, Nishimura YK, Sugane H, Hosoda H, Imai RI, Nakaoka Y, et al. Acute left circumflex coronary artery occlusion: diagnostic problems of initial electrocardiographic changes. Circ Rep 2022;4(10):482-489. https://doi.org/10.1253/circrep.CR-22-0044

22. Lai Y, Chen Y, Wu K, Chen Y. Validation of the diagnosis and triage algorithm for acute myocardial infarction in the setting of left bundle branch block. Am J Emerg Med 2020;38(12):2614-2619. https://doi.org/10.1016/j.ajem.2020.03.024

23. Lindow T, Mokhtari A, Nyström A, Koul S, Smith S, Ekelund U. Comparison of diagnostic accuracy of current left bundle branch block and ventricular pacing ECG criteria for detection of occlusion myocardial infarction. Int J Cardiol 2024;395:131569. https://doi.org/10.1016/j.ijcard.2023.131569

24. Liu C, Yang F, Hu Y, Zhang J, Li X, Guo Z, et al. Combining electrocardiographic criteria for predicting acute total left main coronary artery occlusion. Front Cardiovasc Med 2022;9:936687. https://doi.org/10.3389/fcvm.2022.936687

25. McMahon R, Siow W, Bhindi R, Soo Hoo S, Figtree G, Hansen P, et al. Left bundle branch block without concordant ST changes is rarely associated with acute coronary occlusion. Int J Cardiol 2013;167(4):1339-1342. https://doi.org/10.1016/j.ijcard.2012.04.014

26. Meng Y, Guo S, Li J, Ma J, Li S. Special electrocardiographic manifestations of culprit vessel occlusion in patients with non-ST elevation myocardial infarction. J Clin Cardiol 2022;38(11):876-881. https://doi.org/10.13201/j.issn.1001-1439.2022.11.007

27. Meyers HP, Limkakeng A, Jaffa E, Patel A, Theiling B, Rezaie S, et al. Validation of the modified Sgarbossa criteria for acute coronary occlusion in the setting of left bundle branch block: a retrospective case-control study. Am Heart J 2015;170(6):1255-1264. https://doi.org/10.1016/j.ahj.2015.09.005

28. Meyers HP, Bracey A, Lee D, Lichtenheld A, Li W, Singer D, et al. Ischemic ST-segment depression maximal in V1-V4 (versus V5-V6) of any amplitude is specific for occlusion myocardial infarction (versus nonocclusive ischemia). J Am Heart Assoc 2021;10(23):e022866. https://doi.org/10.1161/JAHA.121.022866

29. Meyers H, Simančík F, Herman R, Rafajdus A, Frick W, Nunes de Alencar J, et al. Hyperacute T waves are specific for occlusion myocardial infarction, even without diagnostic ST-segment elevation. JACC Adv 2025;4(10P2):102120. https://doi.org/10.1016/j.jacadv.2025.102120

30. Meyers HP, Sharkey SW, Herman R, de Alencar JN, Shroff GR, Frick WH, et al. Failure of standard contemporary ST-elevation myocardial infarction electrocardiogram criteria to reliably identify acute occlusion of the left anterior descending coronary artery. Eur Heart J Acute Cardiovasc Care 2025;14(7):403-411. https://doi.org/10.1093/ehjacc/zuaf037

31. Pride Y, Tung P, Mohanavelu S, Zorkun C, Wiviott S, Antman E, et al. Angiographic and clinical outcomes among patients with acute coronary syndromes presenting with isolated anterior ST-segment depression: a TRITON-TIMI 38 (Trial to Assess Improvement in Therapeutic Outcomes by Optimizing Platelet Inhibition with Prasugrel) substudy. JACC Cardiovasc Interv 2010;3(8):806-811. https://doi.org/10.1016/j.jcin.2010.05.012

32. Schmitt C, Lehmann G, Wailersbacher M, Wailersbacher K, Schmieder S, Karch M, et al. Problems of electrocardiographic diagnosis of occlusion of the left circumflex coronary artery. Dtsch Med Wochenschr 2001;126(45):1257-1260. https://doi.org/10.1055/s-2001-18328

33. Smith S, Dodd K, Henry T, Dvorak D, Pearce L. Diagnosis of ST-elevation myocardial infarction in the presence of left bundle branch block with the ST-elevation to S-wave ratio in a modified Sgarbossa rule. Ann Emerg Med 2012;60(6):766-776. https://doi.org/10.1016/j.annemergmed.2012.07.119

34. Tang W, Xu J, Cheng F, Liu T, Lin Z, Chen B, et al. Coronary angiographic features of de Winter syndrome: more than just occlusion of the left anterior descending artery. Ann Noninvasive Electrocardiol 2024;29(6):e70029. https://doi.org/10.1111/anec.70029

35. Verouden N, Koch K, Peters R, Henriques J, Baan J, Van Der Schaaf R, et al. Persistent precordial “hyperacute” T-waves signify proximal left anterior descending artery occlusion. Heart 2009;95(20):1701-1706. https://doi.org/10.1136/hrt.2009.174557

36. Vives-Borrás M, Moustafa AH, Álvarez-García J, Ferrero-Gregori A, Balcells J, García-Picart J, et al. Clinical and prognostic value of the electrocardiogram in patients with acute occlusion of the left circumflex coronary artery. Am J Cardiol 2017;120(9):1487-1494. https://doi.org/10.1016/j.amjcard.2017.07.038

37. Wall J, White L, Lee A. Novel ECG changes in acute coronary syndromes. Would improvement in the recognition of “STEMI-equivalents” affect time until reperfusion? Intern Emerg Med 2018;13(2):243-249. https://doi.org/10.1007/s11739-016-1595-3

38. Wei Y, Pei D, Deng J, Sasmita BR, Mao L, Jia F. Clinical and electrocardiographic characteristics in NSTEMI patients with acute total occlusion of culprit left circumflex artery. Ann Noninvasive Electrocardiol 2025;30(3):e70070. https://doi.org/10.1111/anec.70070

39. Wen Y, Guo J, Li P, Zhou S, Yan R, Song J. Clinical features of patients with acute myocardial infarction presenting de Winter pattern on electrocardiogram. Chinese Journal of General Practitioners 2021;20(8):868-872. https://doi.org/10.3760/cma.j.cn114798-20210220-00168

40. Wiśniewski P, Rostoff P, Gajos G, Nessler J, Kruszelnicka O. Predictive value of electrocardiographic ST-segment elevation myocardial infarction equivalents for detecting acute coronary artery occlusion in patients with non-ST-segment elevation myocardial infarction. Kardiol Pol 2019;77(6):624-631. https://doi.org/10.33963/KP.14815

41. Xu J, Wang A, Liu L, Chen Z. The de Winter electrocardiogram pattern is a transient electrocardiographic phenomenon that presents at the early stage of ST-segment elevation myocardial infarction. Clin Cardiol 2018;41(9):1177-1184. https://doi.org/10.1002/clc.23002

42. Yamamoto M, Witsch T, Kubota S, Hara H, Hiroi Y. Diagnostic value of lead aVR in electrocardiography for identifying acute coronary lesions in patients with out-of-hospital cardiac arrest. Resuscitation 2019;142:97-103. https://doi.org/10.1016/j.resuscitation.2019.07.017

**Supplemental Appendix S4. Reported and Unreported Bias**

The most prominent author-reported bias by a significant margin is selection bias due to inclusion criteria limiting cohorts to high-risk patients or those with confirmed coronary occlusion. Variations of this bias were reported as ascertainment bias, spectrum bias, referral bias, or partial verification bias. Survival bias was also frequently reported, as patients who died of presumed ACO before reaching angiography do not appear in study cohorts unless a composite reference standard includes them. Interpreter studies frequently report observer or interpretation bias, as ECG interpretation by experts, AI models, or dedicated researchers with minimal interobserver variability may perform better than interpretation in general clinical practice. The impact of varying intervals between ECG and angiography, or disease progression bias, was conceptually described in a few studies but not named.

Notably, none of our included studies explicitly named incorporation, differential verification, or partial verification bias. The literature we mapped exhibits compound bias in the operational definition of its populations and reference standards:

- **Incorporation bias:** the ECG functions as both the index test and the reference standard for the final STEMI/NSTEMI diagnosis^1, 2^.
- **Differential verification bias:** reference standards differ systematically by index test result, with STEMI criteria triggering emergent angiography and non-STEMI criteria triggering delayed angiography, medical management, and sometimes no angiographic assessment^3, 4^.
- **Disease progression bias:** variable intervals between ECG and angiography allow dynamic changes in occlusion status to affect reference standard findings^4^.
- **Partial verification bias:** some patients do not reach angiographic verification at all, and the exclusion is not random with respect to index test result^1, 4^.

These systematic features of the STEMI/NSTEMI systems of care pathways act as confounders across much of the literature looking at ECG interpretation in ACS, limiting internal validity. The more recent OMI literature engages substantially with these methodological problems.

**Appendix S4 References**

1. Kohn MA, Carpenter CR, Newman TB. Understanding the direction of bias in studies of diagnostic test accuracy. Acad Emerg Med 2013;20(11):1194-1206. https://doi.org/10.1111/acem.12255

2. McLaren J, de Alencar JN, Aslanger EK, Meyers HP, Smith SW. From ST-segment elevation MI to occlusion MI: the new paradigm shift in acute myocardial infarction. JACC Adv 2024;3(11):101314. https://doi.org/10.1016/j.jacadv.2024.101314

3. Kea B, Hall MK, Wang R. Recognising bias in studies of diagnostic tests part 2: interpreting and verifying the index test. Emerg Med J 2019;36(8):501-505. https://doi.org/10.1136/emermed-2019-208447

4. Whiting PF, Rutjes AWS, Westwood ME, Mallett S, QUADAS-2 Steering Group. A systematic review classifies sources of bias and variation in diagnostic test accuracy studies. J Clin Epidemiol 2013;66(10):1093-1104. https://doi.org/10.1016/j.jclinepi.2013.05.014
